# An Optimized Particle Swarm Algorithm for High-Precision Camera Calibration with Enhanced Wide-Angle Distortion Correction

**DOI:** 10.3390/s26165076

**Published:** 2026-08-10

**Authors:** Qingqing Ji, Zhaoxin Li, Min Shi, Dengming Zhu, Qiao Duan, Yaxuan Liu, Yaotong Wang, Zhaoqi Wang

**Affiliations:** 1 University of Chinese Academy of Sciences, Beijing 100049, China; jiqingqing20b@ict.ac.cn; 2 Institute of Computing Technology, Chinese Academy of Sciences, Beijing 100190, China; zqwang@ict.ac.cn; 3 Agricultural Information Institute, Chinese Academy of Agricultural Sciences, Beijing 100081, China; cszli@hotmail.com; 4 Key Laboratory of Agricultural Big Data, Ministry of Agriculture and Rural Affairs, Beijing 100081, China; 5 School of Control and Computer Engineering, North China Electric Power University, Beijing 102206, China; shi_min@ncepu.edu.cn; 6 Taicang-CAS Institute of Information and Technology, Taicang 215400, China; 7 School of Languages and Communication Studies, Beijing Jiaotong University, Beijing 100044, China; 23121625@bjtu.edu.cn; 8 School of Mathematics Statistics and Mechanics, Beijing University of Technology, Beijing 100124, China; liuyaxuan@emails.bjut.edu.cn; 9 College of Foreign Language, Beijing University of Technology, Beijing 100124, China; 23152102@emails.bjut.edu.cn

**Keywords:** monocular camera, camera calibration, wide-angle camera, particle swarm optimization

## Abstract

Camera calibration is crucial to accurate vision tasks for its establishment of the mapping between 3D space and 2D image space. Traditional calibration methods often suffer from limited accuracy and time-consuming processes. In this study, we propose an automatic guidance system leveraging an improved particle swarm optimization algorithm to achieve fast and high-precision camera calibration. Our system dynamically recommends optimal camera poses for the next calibration image, effectively reducing calibration uncertainty and enhancing accuracy. Furthermore, for wide-angle cameras, we introduce a pre-estimation of distortion coefficients to guide the calibration process, significantly improving the calibration of distortion parameters. Experimental results demonstrate that our method outperforms existing guidance systems, achieving higher calibration accuracy with fewer images and shorter calculation time. The results of camera parameters calibrated by the system are applied to the reconstruction based on point clouds, and can achieve desirable reconstruction effect. The proposed system holds promise for applications in film and television shooting, promoting the development of the industry by reducing calibration errors and equipment debugging time.

## 1. Introduction

Camera calibration establishes the geometric mapping between 3D world points and their corresponding 2D image projections. It is widely applied in machine vision navigation [1], 3D reconstruction [2], and film production [3,4,5], serving as a fundamental prerequisite for vision-based tasks. The accuracy and stability of calibration directly affect the quality of data acquired by the camera, and consequently, the performance of subsequent vision tasks [6]. Therefore, improving calibration accuracy and algorithmic reliability is essential for advancing machine vision applications.

Recent studies have approached camera calibration optimization from various perspectives, including parameter adjustment, marker design, marker recognition, and neural network-based methods. Kim et al [7] employed thermal infrared cameras with multi-view geometry to achieve camera calibration [8]. Lu et al. [9] applied the Harris corner detector within Zhang’s calibration framework [10], achieving sub-pixel accuracy for checkerboard corner extraction and consequently improving overall calibration precision. Cai et al. [11] utilized neural networks to learn the mapping between camera and projector images, effectively correcting distortion in projected images for film and television applications.

Peng et al. [12] developed a guidance system based on Zhang’s calibration method [10] that recommends camera poses when the checkerboard target is fixed, enabling rapid calibration of ordinary cameras. While this system simplifies operation and improves calibration accuracy, it suffers from several limitations, including susceptibility to local optima, long computation time, and limited accuracy gains.

On the basis of Peng et al.’s research, this study proposed a system which can guide the poses of checkerboard calibration targets through a nonlinear optimization algorithm and achieve real-time interaction through graphical user interface (GUI). For each new calibration image to be acquired, the optimal pose and the pose of the calibration target were calculated. The advantage of the designed system over that of Peng et al. refers to the fact that a more advanced nonlinear optimization algorithm was used to find the global optimal solution of the next pose, which overcame the problem of easily falling into local optimal solutions and improves calibration accuracy and speed. In addition, the distortion correction of wide-angle cameras received attention in the study. The introduction of the pre-calibration process helped guide the program to complete its calibration.

The main contributions of our calibration system are threefold. First, we employ an improved particle swarm optimization algorithm to determine the optimal next pose of the calibration target, effectively overcoming the local-optima problem of existing methods. Second, for wide-angle cameras, we introduce a distortion pre-estimation module that significantly reduces calibration errors of distortion coefficients. Third, the proposed system supports both ordinary and wide-angle cameras. Experimental results on multiple cameras demonstrate that our method improves calibration accuracy and reduces calibration time compared to existing guidance systems.

To clearly position our work relative to the existing guiding system of Peng et al [12], Table 1 summarizes the key methodological differences between the two approaches. Although both systems share the same high-level objective—reducing calibration uncertainty by recommending the optimal next pose—our method differs fundamentally in four aspects: (1) the optimization algorithm is upgraded from simulated annealing (SA) to a variable-weight particle swarm optimization (PSO) method, which offers faster convergence and enhanced global search capability; (2) our system supports both conventional and wide-angle cameras, whereas the system of Peng et al. is restricted to conventional cameras only; (3) we introduce a distortion pre-estimation module based on convex optimization, which provides accurate initial distortion priors for wide-angle calibration; and (4) our guidance procedure is fully automated and does not require human intervention to escape from local optima.

Apart from the replacement of the optimization algorithm, the scientific contributions of this paper are mainly reflected in three aspects. First, this paper proposes a pose guidance strategy based on variable-weight PSO, which theoretically guarantees superior global optimization performance compared with simulated annealing and provides rigorous demonstration for inertia-weight scheduling, representing a novel application of adaptive swarm intelligence algorithms for camera calibration guidance tasks. Second, this paper introduces a single-image distortion pre-estimation framework based on convex optimization to deliver effective prior knowledge for wide-angle camera calibration, and such problem formulation has never been covered in previous guidance systems. Third, abundant experiments are conducted to establish quantitative evidence verifying that the proposed PSO AUTO guided calibration achieves higher calibration accuracy with fewer images and shorter computation time, and practical application value is further validated via 3D reconstruction experiments.

## 2. Related Work

Camera calibration has remained a research hotspot in computer vision for decades, and can be broadly categorized into four main approaches.

Traditional calibration methods are largely built upon Zhang’s planar checkerboard framework [10], with many studies introducing intelligent optimization algorithms [13,14,15,16] to refine camera parameters. For instance, Huang et al. [17] developed spatially structured-light templates using color coding to mitigate the impact of target flatness on calibration results, combining graph theory with bundle adjustment to reduce noise sensitivity. However, these methods are sensitive to target flatness, size, and corner-point quality [18], which limits their practicality in non-ideal conditions. Self-calibration methods derive from the seminal work of Maybank and Faugeras [19], with subsequent developments based on Kruppa equations [20,21] and the absolute quadric or plane at infinity [22,23]. These methods typically require objects with known shapes or line segments to perform calibration. Zhu et al. [24] combined optimal polarization information with Zhang’s method to address the problem of highlighted regions in calibration images. Nevertheless, self-calibration methods have stringent requirements for lighting conditions and target surfaces, limiting their practicality.

Active vision-based calibration methods obtain constraints by controlling the camera to perform specific motions on an active vision platform [25,26]. Yuan et al. [27] established the correspondence between light rays and pixels by analyzing the relative pose between cell arrays and principal points, achieving accurate calibration for line-scan cameras. Deng et al. [28] proposed a method using ten infrared horizon sensors to calibrate infrared horizon cameras. While these methods have relatively simple algorithms and can handle unknown complex scenarios, they require precise motion trajectory control and are sensitive to noise and initial value selection, leading to limited robustness [29]. Moreover, obtaining accurate motion parameters in practice is difficult, which restricts their application scenarios.

Neural network-based calibration methods [30,31,32,33] directly learn the mapping from 2D inputs to 3D outputs without explicitly modeling intrinsics, extrinsics, or distortion. Hu et al. [30] applied a BP neural network with improved genetic operators to binocular camera calibration. Zhang et al. [31] optimized BP neural networks to simplify the calibration process and improve accuracy. Butt et al. [32] employed a multi-task learning approach for joint estimation of intrinsics and extrinsics using a novel camera projection loss. Munoz et al. [33] used genetic algorithms to optimize BP neural networks for improved accuracy. Although these methods can achieve high accuracy and exhibit strong fault tolerance, their training processes are time-consuming and they remain susceptible to local optima.

Reference-object-based calibration methods are widely used, due to their simplicity and accuracy. Common reference objects include 1D lines [34] and 2D planar targets with checkerboard, solid circle [35], or concentric circle patterns. Richardson et al. [36,37] adopted policy-based algorithms to select optimal poses from a pre-constructed dataset. Usamentiaga et al. [38] designed a 13-cylinder target that uses laser-line projections to produce elliptical contours for camera pose estimation. Perez et al. [39] developed a calibration marker consisting of two spheres connected by a thin rod to simplify extrinsic calibration in multi-camera environments. Genovese et al. [40] designed a circular-array target with solid circles in a rectangular grid, extending the field of view using plane mirrors for large-scale camera calibration. Qin et al [41] proposed a method for calibrating four fisheye cameras in autonomous driving by iterating between line detection and pose estimation.

In summary, existing studies primarily focus on marker design, parameter calculation, or AI-based optimization, but rarely address the calibration process itself. Consequently, they often suffer from limited accuracy and long computation time. This study improves the calibration process by combining an enhanced optimization algorithm with a pose guidance system, effectively improving accuracy and reducing calibration time. For wide-angle cameras, we introduce a distortion pre-calibration module to address the large calibration errors and time-consuming procedures typically encountered. These contributions make our method suitable for high-precision visual tasks such as 3D reconstruction [42,43], visual perception [44,45], and parameter measurement [46,47,48,49].

The proposed method shares methodological commonality with several recent advances in multi-modal learning and adaptive optimization. YOLO-TCS [50] addresses the issues of large object-scale variations and complex environmental disturbances in traffic sign detection under challenging conditions by employing multi-level feature fusion and a dual-view channel-position attention mechanism, which dynamically adjusts feature representations to adapt to challenges caused by object scale, illumination variations, and viewing perspective. MTLHand [51] adopts a deep convolutional neural network-based multi-task learning framework, embedding soft thresholding as a nonlinear transform layer into an improved ResNet to extract deep hybrid thermal handprint features. Through the synergy of spatial and channel attention, it decomposes the hybrid features into identity-related and time-related components, thereby jointly addressing the tasks of thermal identity recognition and thermal departure time estimation. The Deep Soft-Threshold Feature Separation Network [52] further demonstrates the effectiveness of adaptive feature decomposition in infrared handprint recognition by proposing an adaptive soft-threshold activation function to mitigate feature noise, effectively separating identity-related and time-related feature information from infrared images. Specifically, YOLO-TCS dynamically adjusts feature weights via a triple feature fusion module and a dual-view attention mechanism to achieve adaptive detection of multi-scale traffic signs—its core idea lies in allocating attention weights dynamically, according to the input image content. MTLHand decomposes hybrid features into identity-related and time-related components via a soft-threshold transform layer and spatial/channel attention—its core lies in achieving dynamic feature disentanglement and recombination through attention mechanisms. The Deep Soft-Threshold Feature Separation Network addresses feature noise via an adaptive soft-threshold activation function—its core lies in adjusting the denoising threshold dynamically based on feature distribution.

A common rationale underlying the above methods is the dynamic adjustment of feature weights or attention to balance multiple competing objectives, whether detecting multi-scale objects, disentangling identity features from temporal features, or balancing global exploration and local exploitation in optimization. The variable-weight particle swarm optimization strategy proposed in this paper embodies the identical principle: the linearly decreasing inertia weight [53] enables the algorithm to perform global search with a large weight coefficient (w=0.9) in early iterations to ensure population diversity, and to perform refined local search with a small weight coefficient (w=0.4) in later iterations to guarantee convergence accuracy, thereby dynamically adapting to the varying optimization demands at different stages of the calibration process. This conceptual correspondence indicates that the dynamic weighting strategy, which has proven effective in multi-modal detection and recognition tasks, is equally applicable to camera calibration—a problem that also requires a balance among multiple competing objectives, such as minimizing reprojection error while ensuring numerical stability and avoiding local optima. The successful application of this principle across diverse fields, from autonomous driving [50] to forensic analysis [51,52], strongly validates the practicality and generalizability of our method beyond the specific task of camera calibration.

Furthermore, just as MTLHand [51] and the Deep Soft-Threshold Feature Separation Network [52] address the problem of information attenuation caused by thermal feature fading over time through multi-task decomposition strategies, our distortion pre-estimation module (Section 5) restores a reliable initial distortion estimate from a single wide-angle image as a priori knowledge, thereby addressing the problem of distortion information deficiency in wide-angle imaging. This auxiliary pre-estimation procedure shares methodological similarity with the multi-task decomposition strategies in the aforementioned works: both extract critical prior information through a preceding auxiliary task, provide accurate initial constraints for the subsequent main task, and thereby guide the optimization process toward a more accurate solution.

## 3. System Design

**Figure 1:**
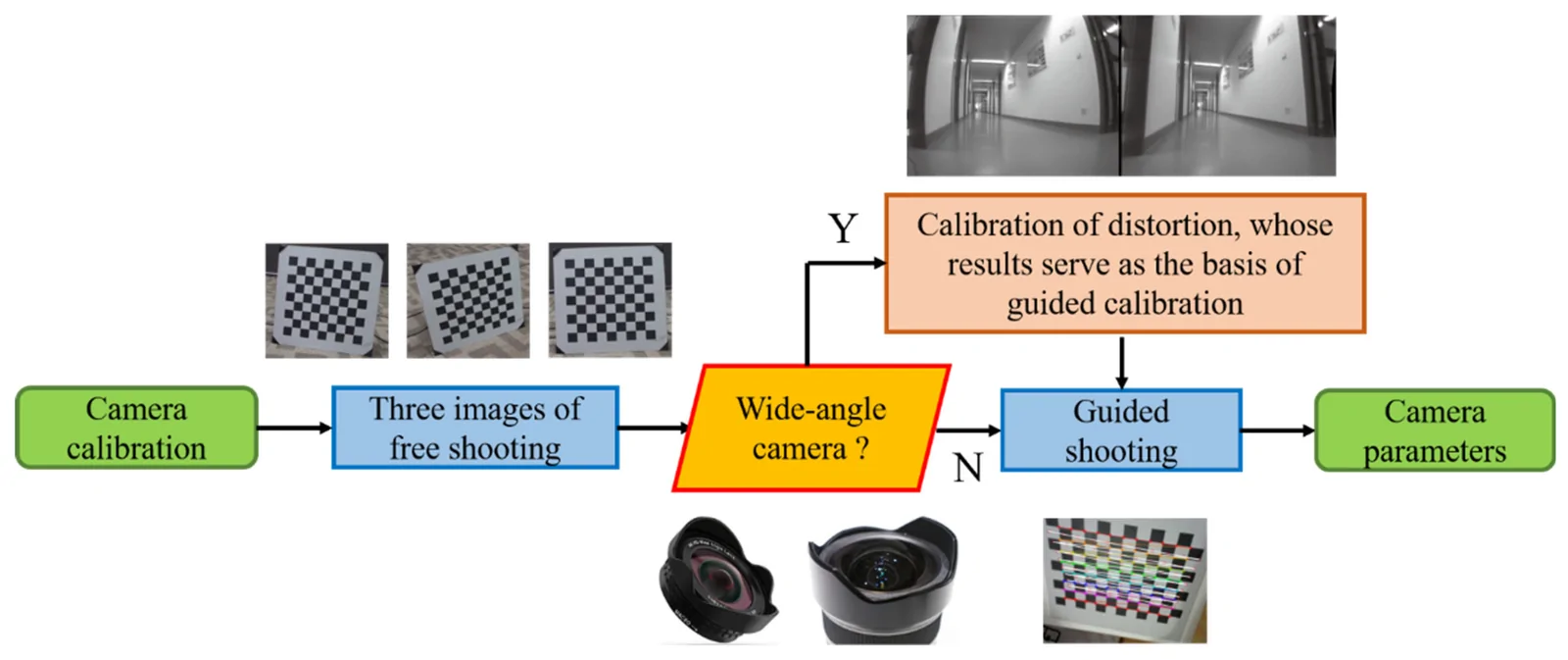
System flowchart.

The designed system is capable of implementing guided calibration for both ordinary and wide-angle cameras. The goal is to provide interactive guidance for the camera calibration to obtain good images. Three freely taken images from the calibration target perform the initial calibration. The system then guides the user to the next best pose through a simple GUI. The specific scheme is shown in Figure 1.

Compared with the guidance system developed by Peng et al. [12], our system introduces three pivotal innovations at the system level. First, the optimization core is upgraded from simulated annealing to the variable-weight PSO algorithm (see Section 4.3 for details), which fundamentally changes the way the next pose is computed. The adaptive inertia-weight mechanism ensures that the algorithm avoids getting trapped in local optima, thereby eliminating the need for manual intervention. Second, we introduce a dedicated distortion pre-estimation module for wide-angle cameras (Section 5). Running prior to the main calibration loop, this module provides a reliable initial estimate of distortion parameters. Conceptually independent of the pose guidance algorithm, it can be enabled or disabled selectively, depending on the camera type. Third, the user interaction paradigm is streamlined. Unlike Peng et al.’s system, which requires users to manually restart optimization when the algorithm stalls, our system offers a fully automated workflow that continuously recommends valid poses until the calibration uncertainty converges to a preset threshold. Together, these system-level improvements, combined with algorithmic enhancements, make our method more robust, efficient, and user-friendly in practical applications.

For the camera calibration task, the system first randomly places the calibration target three times with the pose of the camera fixed (or randomly places the camera three times with the position of the calibration target fixed) and acquires calibration images, determining whether the camera to be calibrated is a wide-angle camera through the analysis of calibration images. If the camera is a wide-angle camera, it enters the distortion pre-estimation module to pre-estimate distortion parameters of the camera and then conduct subsequent guided shooting. If it is an ordinary camera, it directly enters the guided-shooting process. Finally, the calibration images collected by free shooting and guided shooting are used to calculate the camera parameters together.

**Figure 2:**
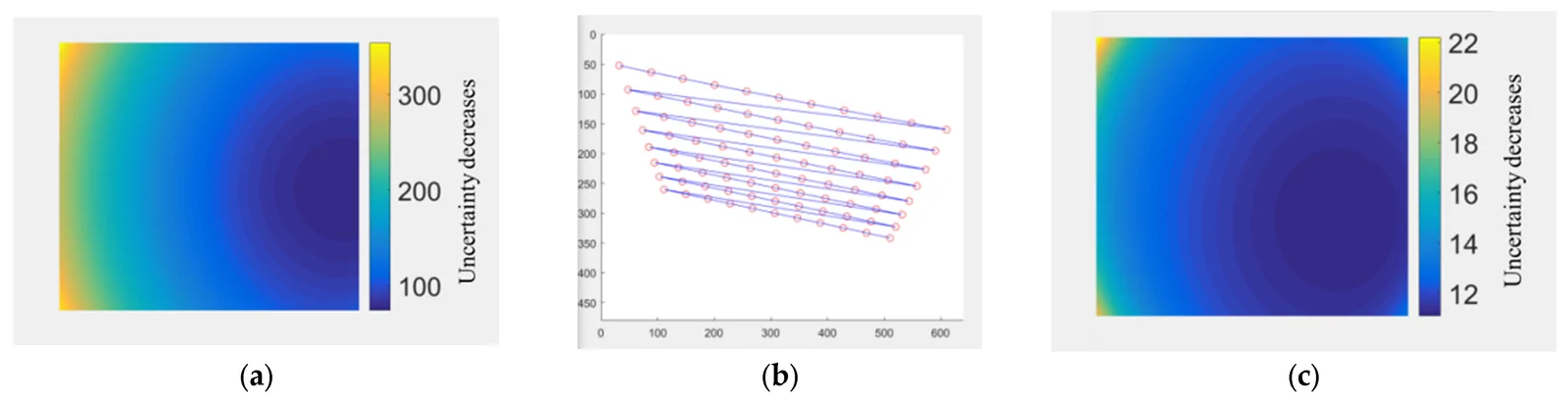
Variation of uncertainty values of the system. (**a**) Uncertainty obtained with the first picture of the marker is taken; (**b**) the next optimal pose of the marker under the guidance of the system; (**c**) uncertainty obtained with the picture of the marker is taken according to the guided pose.

When the system in the study recommends the next best pose of the camera with respect to the calibration target, its reliability can be judged intuitively with the consideration of uncertainty. Figure 2 shows the significant change of uncertainty values before and after the pose guided by the system in free-shooting scenarios. When the uncertainty decreases, it can be assumed that the recommended pose can achieve more accurate calibration results. Figure 2a shows the calculated uncertainty of the current camera parameters when the first picture of the marker is taken, and it can be found that the values are large. Figure 2b shows the next best pose of the calibrated marker recommended by the algorithm of the system during the calibration process. Figure 2c shows the calculated uncertainty of intrinsics and extrinsics of the camera after the picture of the marker taken, according to the pose recommended in Figure 2b. It can be found that a significant decrease was achieved.

## 4. Methodology

### 4.1. Camera Calibration Scheme

The camera is modeled by two local projection functions $x=q_{x}\left(\theta ,s\right),\;y=q_{y}\left(\theta ,s\right)$, which map the 3D point S in the camera coordinate system onto x and y in the image coordinate system. For example, the standard three-parameter pinhole model includes focal length f and main point (u, v), namely $\theta ={\left(f,u,v\right)}^{⊺}$. The local projection Function (1) is thus obtained.

(1) $$q_{x}(\theta ,s)\;=\;u+f\frac{S_{1}}{S_{3}}q_{y}(\theta ,s)\;=\;u+f\frac{S_{2}}{S_{3}}$$

The camera pose is given by the vector Π of 6 intrinsics and extrinsics. It can be expressed as $\Pi ={\left(t_{1},t_{2},t_{3},\alpha ,\beta ,\gamma \right)}^{⊺}$, among which $t={\left(t_{1},t_{2},t_{3}\right)}^{⊺}$ is a displacement matrix. Three angles define a rotation matrix, namely the product of rotations about three coordinate axes: $R=R_{z}\left(\gamma \right)R_{y}\left(\beta \right)R_{x}\left(\alpha \right)$. R and t map the 3D point Q from the world coordinate system to the local coordinate system, according to Equation (2):

(2) S=RQ+t

Therefore, a camera with the pose Π is represented by two global projection functions of Equation (3) $p_{x}$ and Equation (4) $p_{y}$.

(3) $$p_{x}\left(\theta ,\Pi ,Q\right)=q_{x}\left(\theta ,RQ+t\right)=q_{x}\left(\theta ,s\right)$$

(4) $$p_{y}\left(\theta ,\Pi ,Q\right)=q_{y}\left(\theta ,RQ+t\right)=q_{y}\left(\theta ,s\right)$$

Since this study uses the checkerboard as the reference object of calibration, the 3D point Q is predetermined and its corresponding Z coordinate $Q_{3}$ is set to 0. Now we consider m images of the target consisting of n calibration points. Thus, the calibration input is image point $\left(x_{ij},y_{ij}\right)$ of i=1…m and j=1…n, which are detected by the arbitrary corner-detection method, such as the find Chessboard Corners function of Open CV. For the sake of explanation, it is assumed here that all points are visible in all images, though it is not required in reality.

More optimal camera calibration requires a non-linear and simultaneous optimization of all intrinsics and extrinsics (bundle adjustment). This allows a minimum value of geometric reprojection error.

(5) $$\underset{\Theta ,\left\{\Pi_{i}\right\}}{\min}\sum_{i,j}{\left(x_{ij}-p_{x}\left(\Theta ,\Pi_{i},Q_{j}\right)\right)}^{2}+{\left(y_{ij}-p_{y}\left(\Theta ,\Pi_{i},Q_{j}\right)\right)}^{2}$$

Typically, the calibration process is optimized by a local nonlinear least-square optimizer, such as Levenberg–Marquardt. The system designed in this study is independent of the optimizer used; all it requires is to compute the partial derivatives in Equation (5) in the solution( see below).

It is assumed that m images are obtained and intrinsics and poses are estimated from those images by solving Equation (5). Now the goal is to calculate the optimal pose for the next calibration image so that it can minimize the estimated uncertainty of the intrinsics.

Now we consider the Jacobian matrix J derived by the cost Function (5) at the estimated parameters. The matrix J contains the partial derivatives of the residuals of the cost function, that is ${\hat{x}}_{ij}=x_{ij}-p_{x}\left(\Theta ,\Pi_{i},Q_{j}\right)$ and ${\hat{y}}_{ij}=x_{ij}-p_{y}\left(\Theta ,\Pi_{i},Q_{j}\right)$. J comprises a line of each residual. Its columns are usually arranged in groups; for instance, the first group contains partial derivatives with respect to the intrinsic Θ and the subsequent groups are the derivatives with respect to the extrinsics of successive images. Therefore, the high-order sparse form for J is represented by Equation (6) (here, k intrinsics are assumed).

(6) $$J=\left(\begin{array}{ccccc} A_{1} & B_{1} & 0 & \ldots & 0\\ A_{2} & 0 & B_{2} & \ldots & 0\\ \vdots & \vdots & \vdots & \ddots & \vdots \\ A_{m} & 0 & 0 & \ldots & B_{m} \end{array}\right)$$

$$A_{i}=\left(\begin{array}{ccc} \frac{\partial {\hat{x}}_{i1}}{\Theta_{1}} & \ldots & \frac{\partial {\hat{x}}_{i1}}{\Theta_{k}}\\ \frac{\partial {\hat{y}}_{i1}}{\Theta_{1}} & \ldots & \frac{\partial {\hat{y}}_{i1}}{\Theta_{k}}\\ \vdots & \ddots & \vdots \\ \frac{\partial {\hat{x}}_{in}}{\Theta_{1}} & \ldots & \frac{\partial {\hat{x}}_{in}}{\Theta_{k}}\\ \frac{\partial {\hat{y}}_{in}}{\Theta_{1}} & \ldots & \frac{\partial {\hat{y}}_{in}}{\Theta_{k}} \end{array}\right)B_{i}=\left(\begin{array}{ccc} \frac{\partial {\hat{x}}_{i1}}{\Pi_{i1}} & \ldots & \frac{\partial {\hat{x}}_{i1}}{\Pi_{i6}}\\ \frac{\partial {\hat{y}}_{i1}}{\Pi_{i1}} & \ldots & \frac{\partial {\hat{y}}_{i1}}{\Pi_{i6}}\\ \vdots & \ddots & \vdots \\ \frac{\partial {\hat{x}}_{in}}{\Pi_{i1}} & \ldots & \frac{\partial {\hat{x}}_{in}}{\Pi_{i6}}\\ \frac{\partial {\hat{y}}_{in}}{\Pi_{i1}} & \ldots & \frac{\partial {\hat{y}}_{in}}{\Pi_{i6}} \end{array}\right)$$

Among them, $A_{i}$ is a matrix of the size 2n×k, containing partial derivatives of residuals with respect to k intrinsics. $B_{i}$ is a matrix of the size $B_{i}$, containing partial derivatives with respect to extrinsics. Now, the so-called information matrix is represented as $J^{⊺}J$:

(7) $$J^{⊺}J=\left(\begin{array}{ccccc} \sum_{i}A_{i}^{⊺}A_{i} & A_{1}^{⊺}B_{1} & A_{2}^{⊺}B_{2} & \ldots & A_{m}^{⊺}B_{m}\\ B_{1}^{⊺}A_{1} & B_{1}^{⊺}B_{1} & 0 & \ldots & 0\\ B_{2}^{⊺}A_{2} & 0 & B_{2}^{⊺}B_{2} & \ldots & 0\\ \vdots & \vdots & \vdots & \ddots & \vdots \\ B_{m}^{⊺}A_{m} & 0 & 0 & \ldots & B_{m}^{⊺}B_{m} \end{array}\right)$$

Similar to J, $J^{⊺}J$ is highly sparse. More importantly, its inverse ${\left(J^{⊺}J\right)}^{-1}$ provides estimation for an estimated covariance matrix of intrinsics and extrinsics. For camera calibration, only the covariance matrix of intrinsics is concerned, namely the k×k submatrix in the top left corner of ${\left(J^{⊺}J\right)}^{-1}$. Because of the special structure of ${\left(J^{⊺}J\right)}^{-1}$, it can be calculated effectively, enabling

$$\begin{array}{c} U=\sum_{i}A_{i}^{⊺}A_{i} \end{array}$$

$$\begin{array}{c} V=diag\left(B_{1}^{⊺}B_{1},\cdots ,B_{m}^{⊺}B_{m}\right)\\ W=\left(A_{1}^{T}B_{1}\;\cdots \;A_{m}^{T}B_{m}\right) \end{array}$$

Then,

(8) $$J^{⊺}J=\left(\begin{array}{cc} U & W\\ W^{⊺} & V \end{array}\right)$$

Given the inspiration from [54], the submatrix in the top left corner of ${\left(J^{⊺}J\right)}^{-1}$ refers to $\sum ={\left(U-{WV}^{-1}W^{⊺}\right)}^{-1}$, namely the inverse of k×k symmetric matrix.

### 4.2. The Scheme of Guided Camera Calibration

Now the problem we need to solve refers to determining the next best pose $\Pi_{m+1}$. It includes the following steps. The Jacobian matrix in Equation (6) is extended to the part corresponding to additional images and its pose is parameterized by $\Pi_{m+1}$. Thus, the coefficients in $A_{m+1}$ and $B_{m+1}$ are the function of $\Pi_{m+1}$. It is certain that y $\Pi_{m+1}$ is also implied in the covariance matrix of intrinsics associated with this extended system. To reduce the uncertainty, Σ is expected to be as “small” as possible by determining $\Pi_{m+1}$ through the reliability of calibration. Inspired by [54], the study chooses to minimize the inverse of k×k matrix. Since the best pose of the next calibration image is expected to be calculated in the whole 3D workspace, the study utilizes the global optimization method.

Simulated annealing is a stochastic optimization algorithm, which is designed from the principle of solid annealing. Simulated annealing often runs from a higher temperature, namely the initial temperature, and with its continuous decrease, the solution in the algorithm tends to be stable. The stable value is a local optimal solution. At the moment, simulated annealing will jump out of this local optimal solution with lesser probability to find the global optimal solution of the objective function. Peng et al. [12] use simulated annealing to carry out the global optimization of the next optimal pose of the calibration image. This method has difficulty jumping out of the local optimal solution when applied to the problem, and requires manual operation to return to the previous local optimal solution before it continues to run.

Although simulated annealing has strong capability of local search, its global search ability is relatively weak and very susceptible to the model parameters. This poses a challenge for the optimization of the optimal pose of the calibration image. In practical applications, the method converges slowly and takes a long time to solve the global optimal solution. When this algorithm is used to predict poses, it often takes about one minute to find the optimal solution for the objective function for each set of calibration images in the best pose. Moreover, the performance of the algorithm is related to the parameter settings. If the cooling rate is slow during the annealing process of the algorithm, it converges slowly; if the cooling rate is too fast, it is likely that the global optimal solution is not obtained and the system cannot continue the calibration. Therefore, the present method has large limitation and requires a more robust optimization algorithm to predict the best pose for the next calibration picture.

### 4.3. Particle Swarm Optimization with Variable Weights

The adoption of the particle swarm optimization (PSO) method over the simulated annealing (SA) method is mainly motivated by three fundamental limitations of the SA method in pose guidance scenarios. First, the SA method relies on a probabilistic acceptance criterion to allow occasional inferior moves for escaping local optima. However, the annealing schedule required by this mechanism is highly problem-dependent, and improper parameter settings can lead to premature convergence or excessively long optimization time. Second, the SA method explores the solution space by randomly perturbing a single candidate solution. Its inherent sequential nature makes it unsuitable for high-dimensional pose optimization problems with large search spaces. Third, the SA method does not exploit any structural information of the objective function, resulting in a slow convergence rate. In the implementation of Peng et al. [12], each pose prediction takes approximately 60 s.

In comparison, the PSO method maintains a population of candidate solutions (particles) to explore the search space simultaneously, achieving parallel exploration and faster convergence. The velocity update rule in the PSO method incorporates the individual optimal experience (cognitive component) of each particle and the global optimal experience (social component) of the swarm, which realizes efficient information-sharing among particles. In addition, the variable-weight strategy Formula (11) provides a principled approach to balance exploration and exploitation without relying on problem-specific parameter tuning. As demonstrated in the experiments of this paper, these advantages translate into significantly reduced prediction time and greatly improved calibration accuracy.

The guidance system for camera calibration designed in this study aims to continuously reduce the average error of reprojection in the process of calibration. In other words, the smaller the value of the objective function Σ, the better it is. Hence, it naturally introduces the particle swarm algorithm to solve the global optimal solution of the objective function.

For each next hypothetical pose we want to examine, we can use this hypothetical pose and the current intrinsics to project the points of the calibration target onto the image plane so that the expected position of each corner point is obtained. Then it can be used to guide the user with that pose.

In order to obtain the next best pose, which means that the optimal camera internal parameters need to be obtained, we need to reduce the expected uncertainty of the camera internal parameters.

To obtain the next best pose means to obtain the optimal intrinsics of the camera. It requires us to reduce the expected uncertainty of the intrinsics, and to find the global optimal solution to the objective function by using the particle swarm algorithm, namely the inverse of k×k symmetric matrix in the top left corner of ${\left(J^{⊺}J\right)}^{-1}$.

In order to improve the applicability of particle swarm optimization, the formula of the particle swarm optimization is transformed into the following ones under the inspiration of the inertia-weight method proposed by Shi and Eberhart [53]:

(9) $$X_{a}^{t+1}=X_{a}^{t}+V_{a}^{t+1}$$

(10) $$V_{a}^{t+1}={w\cdot V}_{a}^{t}+c_{1}r_{1}p{best}_{a}^{t}-X_{a}^{t}+c_{2}r_{2}\left(g{best}_{a}^{t}-X_{a}^{t}\right)$$

In the formula, $c_{1}$ and $c_{2}$ are the learning factors of particles; $r_{1}$ and $r_{2}$ refer to random numbers between 0 and 1. The weight coefficient w is set to control the influence of the velocity of the previous generation on that of the current particle. When w=1, the algorithm reverts to the elementary particle swarm optimization. Reasonable setting of weight coefficients can improve the performance of the algorithm. To this end, Shi and Eberhart proposes the method of linear reduction of weight coefficients:

(11) $$w=w_{max}-\frac{t}{t_{max}}\left(w_{max}-w_{min}\right)$$

In Equation (11), $w_{max}$ is the maximum weight coefficient, $w_{min}$ is the minimum weight coefficient, $t_{max}$ is the maximum number of iterations, and t is the current number of iterations. At the beginning of iteration, a large weight coefficient is used for global search. When a better solution is found, a small weight coefficient is used for local search.

To ensure the reproducibility of our method, Table 2 lists all the key implementation parameters of the variable-weight PSO algorithm.

The initialization and update of particle positions and velocities abide by the following constraints.

The initial position ($X_{a}$) of each particle is assigned a value sampled uniformly at random within the feasible pose space:

(12) $$X_{a}^{0}∼U\left(X_{min},X_{max}\right)\;$$

The velocity ($V_{a}$) of each particle is initialized to a zero vector and clamped to the velocity bounds after each update:

(13) $$V_{a}^{t+1}=\mathrm{clip}\left(V_{a}^{t+1},V_{min},V_{max}\right)$$

Among them, $V_{min}=-0.1\times \left(X_{max}-X_{min}\right)$, $V_{max}=0.1\times \left(X_{max}-X_{min}\right)$.

In the calibration scenario described in this paper, the search boundaries for the pose parameter space $\Pi ={\left(t_{1},t_{2},t_{3},\alpha ,\beta ,\gamma \right)}^{⊺}$ are defined based on the working distance range between the calibration board and the camera. Specifically, the search range for the translation components $t_{1},t_{2},t_{3}$ is set to a cubic region centered at the camera’s optical center with dimensions of ±200 mm (determined by the actual size of the calibration board and the camera’s field of view), while the search range for the rotation components α,β,γ is set to $\pm 90^{\circ }$. The corresponding velocity boundaries are set to ±20 mm per iteration for translation and ±9° per iteration for rotation, preventing particles from deviating beyond physically reasonable pose ranges during the search process.

The selection of these parameters is based on the following considerations: a particle count *N* = 30 strikes a good balance between convergence speed and computational cost, ensuring sufficient population diversity while avoiding excessive computation load (each iteration requires evaluating 30 candidate poses); a maximum number of iterations $t_{max}=200$ is sufficient for the algorithm to converge to a stable solution in most cases; the linearly decreasing inertia weight from 0.9 to 0.4 follows the classic strategy proposed by Shi and Eberhart [53], enabling strong global exploration in early stages and effective local refinement in later stages; the learning factors $c_{1}=c_{2}=1.5$ are widely adopted in PSO literature and effectively balance individual cognitive ability with social collaboration within the swarm. The dual termination criteria—maximum number of iterations and fitness change threshold—ensure that the algorithm neither wastes time due to excessive fixed iterations, nor prematurely terminates, resulting in suboptimal solutions.

After the optimal solution is obtained through particle swarm optimization, the points of calibration object obtained at this time can be projected onto the image plane through the obtained optimal intrinsics, namely the intrinsics of the next best pose. In this way, the image of the best pose can be obtained and used for guiding the user to carry out calibration.

## 5. Distortion Correction of a Single Image Shot by Wide-Angle Cameras

As more than two calibration images must be used to carry out calibration and obtain results in Zhang Zhengyou’s study [31], relevant parameters cannot be obtained by applying this method for a single calibration image. However, due to the particularity of wide-angle cameras, the error of all parameters will be larger, due to the poor calibration results of distortion by means of Zhang Zhengyou’s method. To solve the above problems, the study, before calibration, inputs the initial camera matrix of a single image for calibration to assist the system to calibrate the distortion value of the camera rapidly and accurately, so as to obtain the calibration results closer to real parameters. The specific process of the algorithm is as follows:

The image point in the original radial distorted image shot by wide-angle cameras is called distortion point, and is expressed as $x^{d}$. Its corresponding distortion correction point is expressed as $x^{u}$. The radial deformation center is denoted as e. The three points $x^{d}$, $x^{u}$, and e should be collinear, which enables

(14) $$x^{dT}{\left[e\right]}_{\times }x^{u}=0$$

In the Equation (14), ${\left[e\right]}_{\times }$ represents the skew symmetric matrix of the vector cross product. Let $x^{c}$ be the point on the calibration pattern, and then $x^{c}$ establishes a mapping relationship with its corresponding undistorted image point $x^{u}$ through a 2D mapping relationship. Let $F\propto {\left[e\right]}_{\times }H$, and the basic matrix relation can be obtained as follows:

(15) $$x^{dT}Fx^{c}=0$$

The center of radial distortion can be restored as the left kernel of F. After deduction, the following can be obtained:

(16) $$\left[\begin{array}{ccc} f_{11} & f_{12} & f_{13}\\ f_{21} & f_{22} & f_{23}\\ f_{31} & f_{32} & f_{33} \end{array}\right]\propto \left[\begin{array}{ccc} 0 & -1 & 0\\ 1 & 0 & 0\\ 0 & 0 & 0 \end{array}\right]\left[\begin{array}{ccc} h_{11} & h_{12} & h_{13}\\ h_{21} & h_{22} & h_{23}\\ h_{31} & h_{32} & h_{33} \end{array}\right]=\left[\begin{array}{ccc} {-h}_{21} & -h_{22} & -h_{23}\\ h_{11} & h_{12} & h_{13}\\ 0 & 0 & 0 \end{array}\right]$$

In [55], convex optimization method is used to replace the least square method in [56] to solve the Equation (16), and a better solution is obtained. This study thus adopts this method.

**Figure 3:**
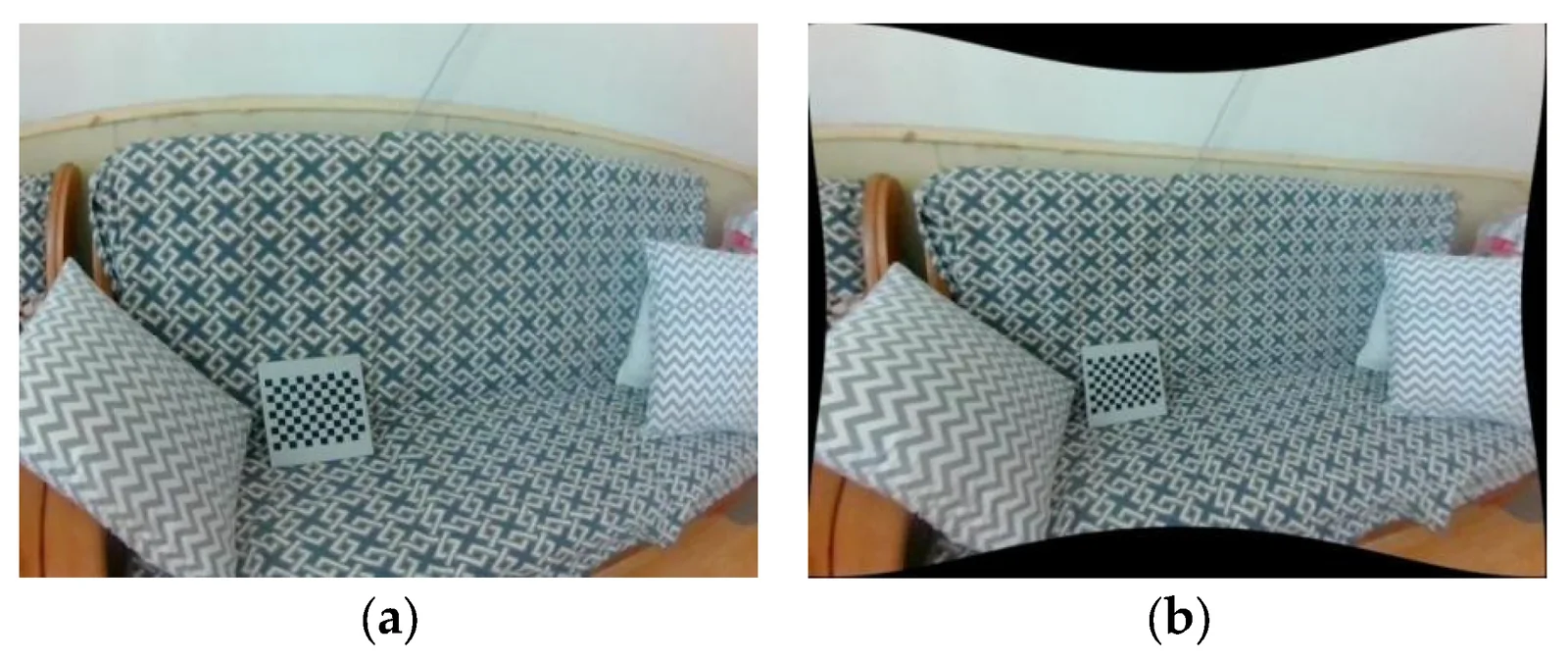
Distortion correction of a single image. (**a**) The image shot by the wide-angle camera; (**b**) effect of calibration of distortion.

The system obtains anti-distortion by means of the method, as shown in Figure 3. It can obtain the coordinates of the center point of distortion in the camera, the focal length, and other information through a single calibration image. These data are introduced into the calibration process of the guidance system in this study as prior knowledge. It can assist the CalibrateCamera function in the calibration system to calibrate distortion parameters of the camera, so as to obtain better results.

## 6. Results and Evaluation

The results of manual calibration and guided calibration were compared, to evaluate the effectiveness of the guidance system for calibration. It should be noted that in the optimization process, all corner points should be within the camera’s field of view when pictures used for calibration are shot, otherwise the loss of optimization is set to a very large value. In Section 6.1, data collection was firstly introduced. In Section 6.2, the calibration results of the designed system and other methods, including focal length and distortion parameters, were compared to analyze advantages and disadvantages of each method. Finally, in Section 6.3, with the usage of calibration results, evaluation of the method in this study was conducted through pose estimation and reconstruction based on point clouds. The method was comprehensively evaluated by the analysis of trial data.

### 6.1. Data Preparation

To evaluate the proposed algorithm in this study, MATLAB 2013a was used to simulate the camera calibration with fixed parameters. The system trial equipment has a processor of Intel i7, RAM of 8 GB, discrete graphics card GTX 1050, and the operating system of Windows 11.

First of all, the data set used for calibration was collected by a diffuse reflection calibration target with 12 × 9 corner points. The calibration target used glass as the substrate and was sprayed with alumina membrane, having the characteristics of a small coefficient of thermal expansion, high precision, high hardness and strong corrosion resistance; its precision could reach μm level. Compared with the calibration target projected by LCD, it can obtain the size of checkerboard more accurately. Because of the ink, the self-made calibration target is prone to have unclear corner points and uneven paper. Therefore, the diffuse reflection calibration target can achieve a better effect of calibration.

Evaluation of accuracy. The study mainly compared the camera calibration results obtained from random images and those obtained from images taken with Peng’s method (SA) and our method (PSO), which were divided into considering and ignoring autocorrelation matrices described in the previous section. To this end, the process of trial is as follows: three initial random images were created, based on which five schemes for obtaining results of camera calibration were established:

- Take images of random calibration on the basis of experience;
- Obtain 17 images suggested by SA, leading to 3 + 17 = 20 images;
- Obtain 17 images suggested by SA AUTO;
- Obtain 17 images suggested by PSO, leading to 3 + 17 = 20 images;
- Obtain 17 images suggested by PSO AUTO

In this study, the ordinary camera adopted was C270i camera of Logitech (Logitech International S.A., Lausanne, Switzerland), which had a resolution of 640 × 480 and a field of view of about 60°. The wide-angle camera adopted was the X30 camera of VISHINSGAE (Sichuan Weixin Vision Technology Co., Ltd., Chengdu, China), which had a resolution of 1080 P and a field of view of about 150°. The resolution of pictures was set to 640 × 480 when they were shot.

**Figure 4:**
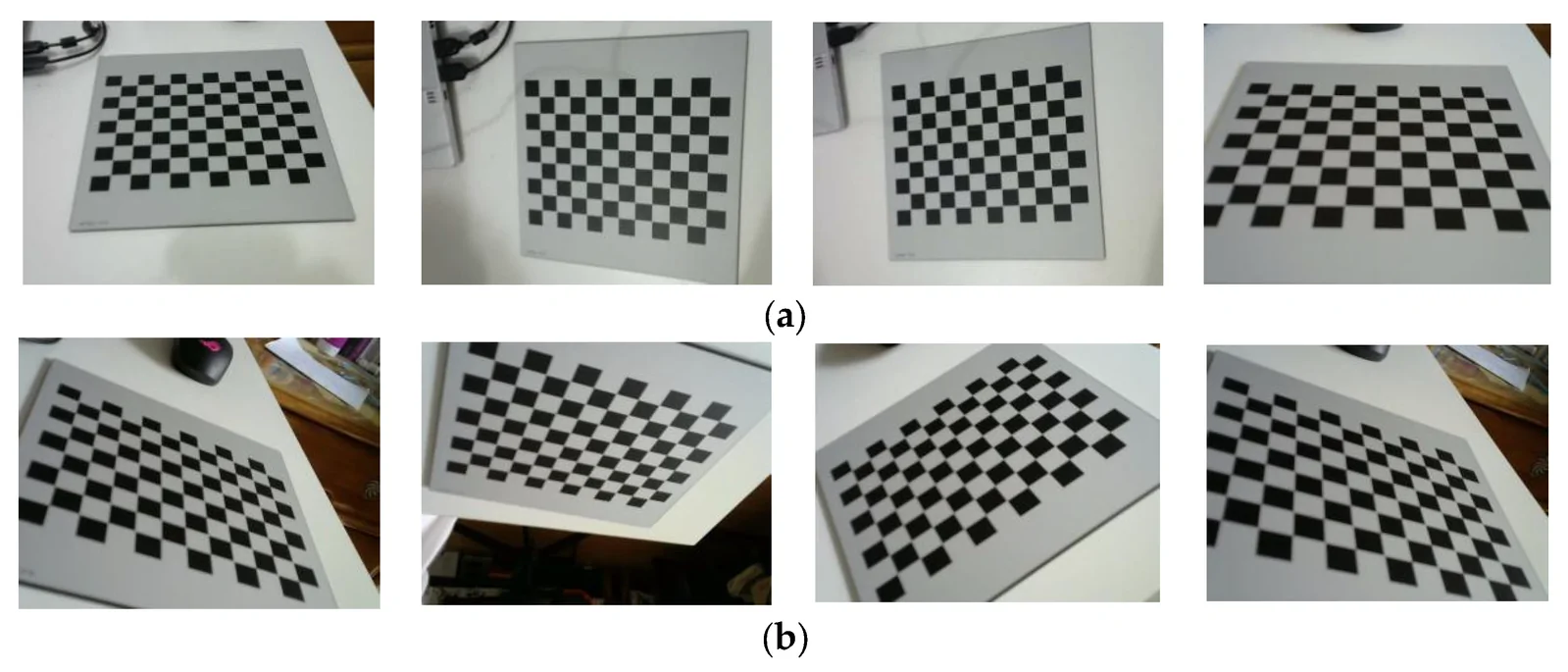
Part of collected data of calibration images of the ordinary camera. (**a**) Images of the calibration target taken by the user independently. (**b**) Images of the calibration target taken with the guidance system.

As shown in Figure 4, both groups were images taken by the calibration target. Group (a) includes images taken by the user independently, and group (b) includes images taken with the guidance system.

We calibrated the camera parameters by shooting multiple random calibration images, and conducted 30 rounds of trials for the ordinary camera and the wide-angle camera, respectively. Finally, the obtained real focal length of the ordinary camera was f=813.21 mm, and the real distortion coefficients were $k_{1}=0.085$ and $k_{2}=0.060$. The real focal length of the wide-angle camera was f=431.00 mm, and the real distortion coefficients were $k_{1}=-0.362$ and $k_{2}=0.115$.

To guarantee the rigor and repeatability of experiments, all calibration tests follow the standardized procedure described below. Each trial commences with three identical initial freely captured images. Specifically, with the calibration board fixed, the camera captures images from three distinct random poses to guarantee clear visibility and reasonable spatial distribution of checkerboard corners on the calibration board. All comparative approaches (the free-shot method, SA AUTO method, and the proposed PSO AUTO method in this paper) adopt these three identical initial images for initial calibration.

For guided calibration methods (the SA AUTO method and the proposed PSO AUTO method), the system independently calculates the optimal next camera pose, according to the real-time calibration results. Afterwards, an operator precisely adjusts the position of the camera or calibration board following the recommended pose to acquire new calibration images. Within each trial, the SA AUTO method and the proposed PSO AUTO method independently generate the entire subsequent guided image sequence (3 initial images + 12 guided images), enabling a fair comparison between the two methods under identical initial conditions.

To account for the randomness embedded in the SA algorithm and PSO algorithm, each group of comparative experiments is repeated for 30 independent trials with distinct random seeds. The algorithm is reinitialized in every trial, and the full calibration pipeline is executed from scratch (starting from the three initial images and acquiring guided images incrementally, until the total number of guided images reaches 12). Final experimental results are presented as the average values of the 30 repeated trials. The reported standard deviation allows readers to evaluate the stability of each method under varied random initialization.

All images are acquired under identical physical conditions: the same calibration board (12 × 9 corner diffuse reflection calibration board), consistent working distance range, and uniform illumination (an ordinary indoor fluorescent lamp, illuminance of 300–500 lux), so as to achieve a fair comparison among different algorithms.

### 6.2. Comprehensive Evaluation

#### 6.2.1. Comprehensive Evaluation of Calibration of Ordinary Cameras

##### Ablation Study: Comparison Between Standard PSO and Variable-Weight PSO

To verify the effectiveness of the linearly decreasing inertia-weight strategy proposed in this paper, camera calibration experiments are conducted under completely consistent conditions, including three identical initial images, twelve identical subsequent guided images, and with the same ordinary Logitech C270i camera. Standard PSO (fixed inertia weight w=1) and variable-weight PSO (inertia weight linearly decreasing from 0.9 to 0.4) are adopted for camera calibration, with 30 independent trials performed for each algorithm.

Table 3 presents the comparative results of the two algorithms. The variable-weight PSO outperforms the standard PSO significantly in all evaluation metrics. The focal length estimation error is reduced from 2.42 to 0.31, corresponding to a decrease of 87.2%. The estimation errors of distortion coefficients $k^{1}$ and $k_{2}$ are decreased by 81.8% and 78.4%, respectively. Meanwhile, the reprojection error drops from 0.1784 pixels to 0.1599 pixels.

In terms of convergence performance, the variable-weight PSO achieves an average convergence iteration number of 112, which is 40.1% lower than the 187 iterations of the standard PSO. Among the 30 trials, the standard PSO falls into local optima in five runs (16.7%), where the deviation between the estimated focal length and the ground truth exceeds 8 mm. In contrast, the variable-weight PSO encounters falls into local optima only once (3.3%). The experimental results verify that the proposed variable-weight strategy is effective in avoiding local optima.

##### An Overall Comparison Between Different Calibration Methods

**Figure 5:**
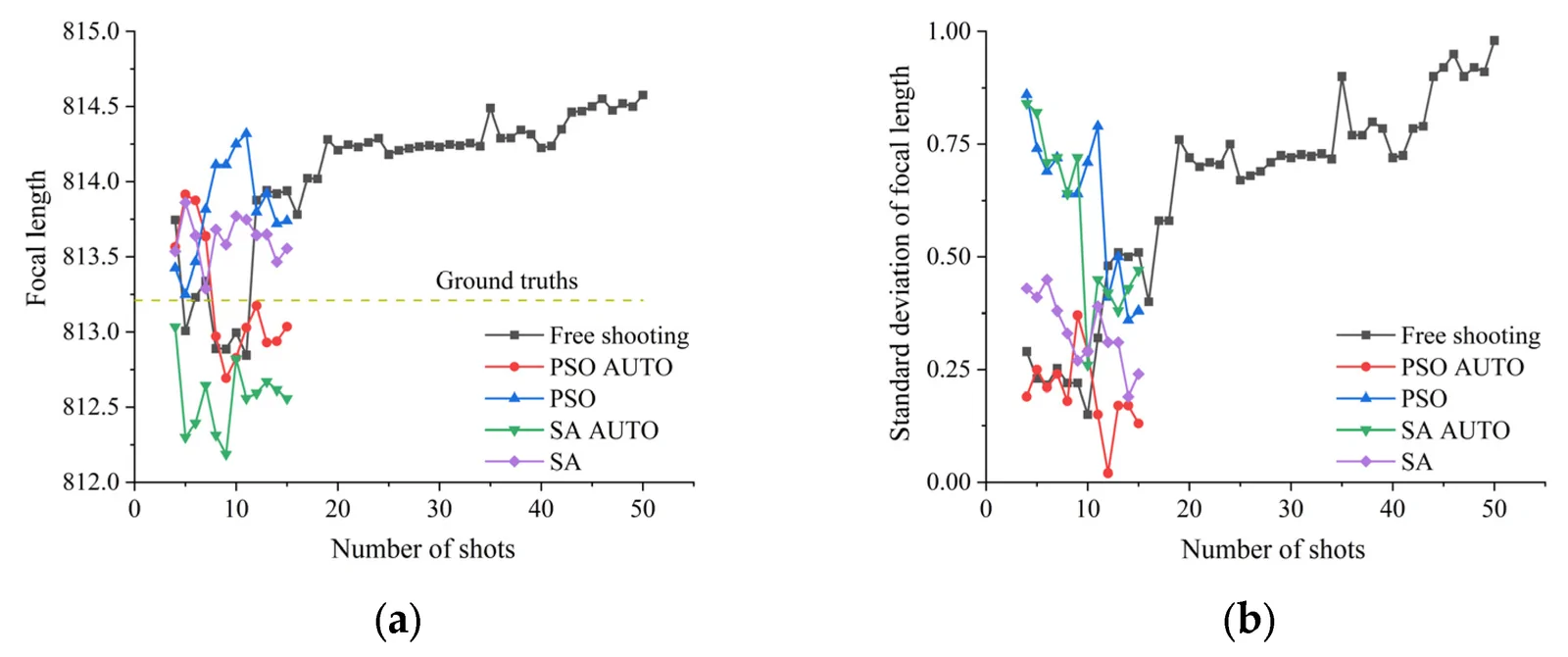
Calibration results of focal length of the ordinary camera under different schemes. (**a**) Calibration results of focal length of the ordinary camera under different schemes. (**b**) Standard deviation of calibrated focal length of the ordinary camera under different schemes.

Figure 5 shows the statistical results and standard deviations of estimated focal length in 20 trials. In Figure 5a, it can be found that the focal length of the ordinary camera obtained by the guidance system in the study was not only closer to the real focal length, but also more accurate than that estimated from random images. For instance, the accuracy of the estimated focal length obtained by PSO AUTO through only three free-shooting images and four guided-shooting images exceeded that obtained by SA AUTO under the same conditions. Meanwhile, it was far better than the accuracy of focal length obtained through 20 random images, which directly proved the effectiveness of the method proposed in the study. In addition, compared with the simple cases using different guidance methods, the results of estimated focal length were more ideal and closer to the real value when autocorrelation matrices of feature points were considered. In Figure 5b, from the changes of the standard deviation of focal length, the standard deviation of the calibrated focal length of the ordinary camera obtained by free shooting gradually increased and had a large value; the standard deviation of calibrated focal length obtained by SA AUTO and PSO AUTO tended to decrease rapidly, and had a small value.

**Figure 6:**
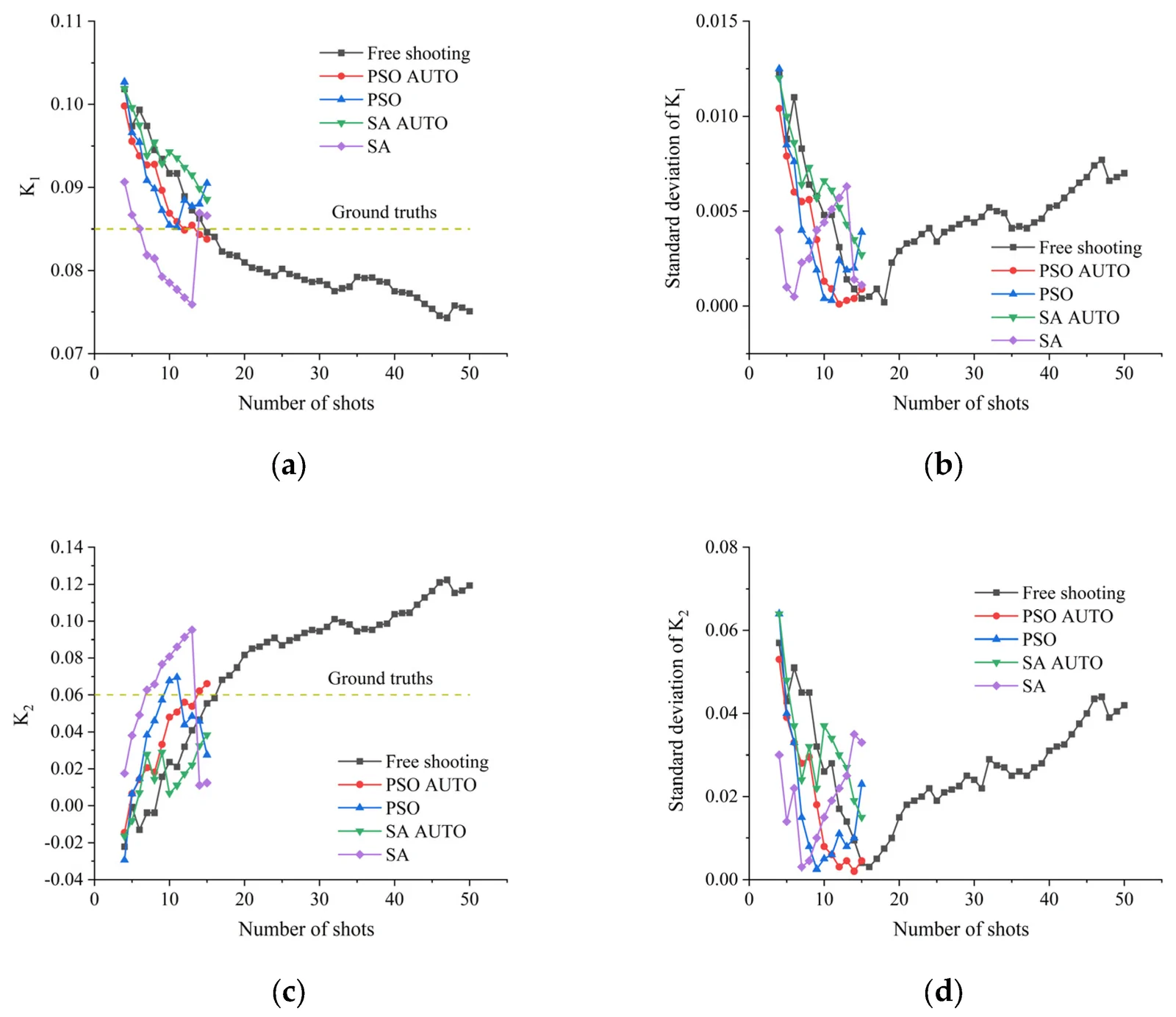
Calibration results of distortion parameters $k_{1}$ and $k_{2}$ of the ordinary camera under different schemes. (**a**) Calibration results of $k_{1}$ of the ordinary camera under different schemes; (**b**) standard deviation of calibrated $k_{1}$ of the ordinary camera under different schemes; (**c**) calibration results of $k_{2}$ of the ordinary camera under different schemes; (**d**) standard deviation of calibrated of $k_{2}$ of the ordinary camera under different schemes.

A certain degree of distortion was manifested in ordinary cameras as well, but the distortion was usually invisible because of small value of distortion parameters. To better evaluate the effect of the guidance algorithm, the calibration results of distortion parameters $k_{1}$ and $k_{2}$ of ordinary cameras were also compared, as shown in Figure 6.

As can be seen from Figure 6a,c, the distortion values $k_{1}$ and $k_{2}$ of the ordinary camera obtained by PSO AUTO were closer to the real values than those obtained by other guidance schemes, and its number of calibration images was smaller. From the changes in standard deviation of distortion coefficient of the ordinary camera in Figure 6b,d, the standard deviation of calibrated distortion coefficient obtained by free shooting gradually increased, representing the fact that the calibration error of distortion coefficient became increasingly larger with the increase in the number of calibration images. However, the standard deviation of calibrated distortion coefficient obtained by SA AUTO and PSO AUTO tended to decrease rapidly, and both eventually approached to zero. It can be further demonstrated that PSO performed better guidance for calibration.

#### 6.2.2. Comprehensive Evaluation of Calibration of Wide-Angle Cameras

##### Ablation Study: Effectiveness Verification of the Distortion Pre-Estimation Module

To verify the effectiveness of the convex-optimization-based single-image distortion pre-estimation module proposed in Section 5, ablation experiments are carried out on a wide-angle camera (VISHINSGAE X30, FOV ≈ 150°). With all other experimental conditions unchanged (identical three initial images and twelve subsequent guided images), the calibration performance with and without the distortion pre-estimation module activated is tested separately.

Table 4 summarizes the comparative results of the two schemes. After activating the distortion pre-estimation module, the focal length-estimation error drops from 5.84 mm to 1.15 mm, representing a reduction of 80.3%. The estimation errors of distortion coefficients ($k_{1}$) and ($k_{2}$) are reduced by 88.9% and 88.2%, respectively. The reprojection error decreases from 2.4231 to 1.7465, a decline of 27.9%. More critically, the distortion center-shift error is reduced from 11.6 pixels to 0.8 pixels, with a reduction rate of 93.1%, which establishes an extremely accurate spatial reference for subsequent distortion correction. 

Further, to analyze the effect of the pre-estimation module on different image regions, we divided the image into a central region (radius ≤ 1/3 of the shorter image side) and an edge region (radius > 1/3 of the shorter image side), following the reviewer’s advice, and calculated the residual errors after distortion correction separately. As shown in Table 5, the error reduction rate of the pre-estimation module is 26.3% for the central region and 59.8% for the edge region. The more prominent improvement in the edge region can be explained as follows: distortion of wide-angle lenses increases nonlinearly, and the distortion magnitude in the edge region (approximately 30–50 pixels) is far larger than that in the central region (approximately 5–8 pixels). The pre-estimation module acquires accurate distortion center coordinates and initial distortion coefficients via convex optimization, which provides critical prior constraints for modeling the strong nonlinear distortion in edge regions. Accordingly, it effectively alleviates the difficulty of distortion fitting for edge areas.

In terms of convergence speed, after enabling the pre-estimation module, the number of images required to reach stable precision (the variation in reprojection error is less than 0.01 pixels) decreases from 9.8 to 6.2, a reduction of 36.7%. Although the pre-estimation module brings an extra computational overhead of approximately 5–6 s (the total prediction time rises from 139.8 s to 145.0 s, with an increment of around 3.7%), the remarkable improvements in calibration accuracy and convergence speed far outweigh this slight time overhead.

The above experimental results fully verify the effectiveness and necessity of the proposed single-image distortion pre-estimation module based on convex optimization for wide-angle camera calibration.

##### The Overall Comparison Between Different Calibration Methods

**Figure 7:**
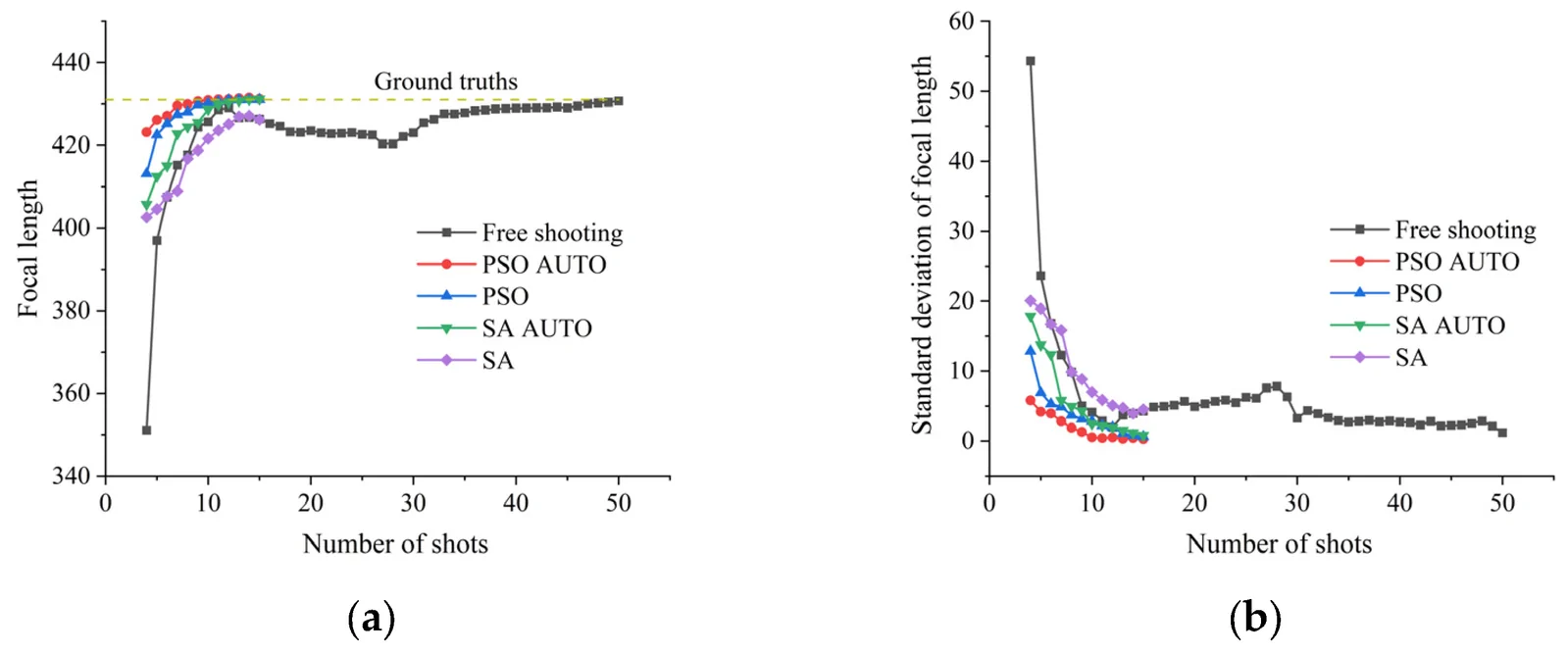
Calibration results of focal length of the wide-angle camera under different schemes. (**a**) Calibration results of focal length of the wide-angle camera under different schemes. (**b**) Standard deviation of calibrated focal length of the wide-angle camera under different schemes.

The estimated focal length of the wide-angle camera under different schemes was analyzed, as shown in Figure 7. It can be found in Figure 7a that all the estimated values were close to the real focal length of the wide-angle camera after a period of operation of different schemes. PSO AUTO had the best effect. Regardless of whether autocorrelation matrices were considered or not, PSO AUTO was better than SA AUTO. The calibration results obtained by free shooting were the worst. This set of trials clearly and directly reflected positive guidance of the PSO AUTO for wide-angle camera calibration in this study. Figure 7b shows the standard deviation of calibrated focal length of the wide-angle camera under different schemes. It can be seen that the standard deviation of calibrated focal length of the wide-angle camera obtained by PSO AUTO was smaller than that obtained by SA AUTO, and the data was more concentrated.

**Figure 8:**
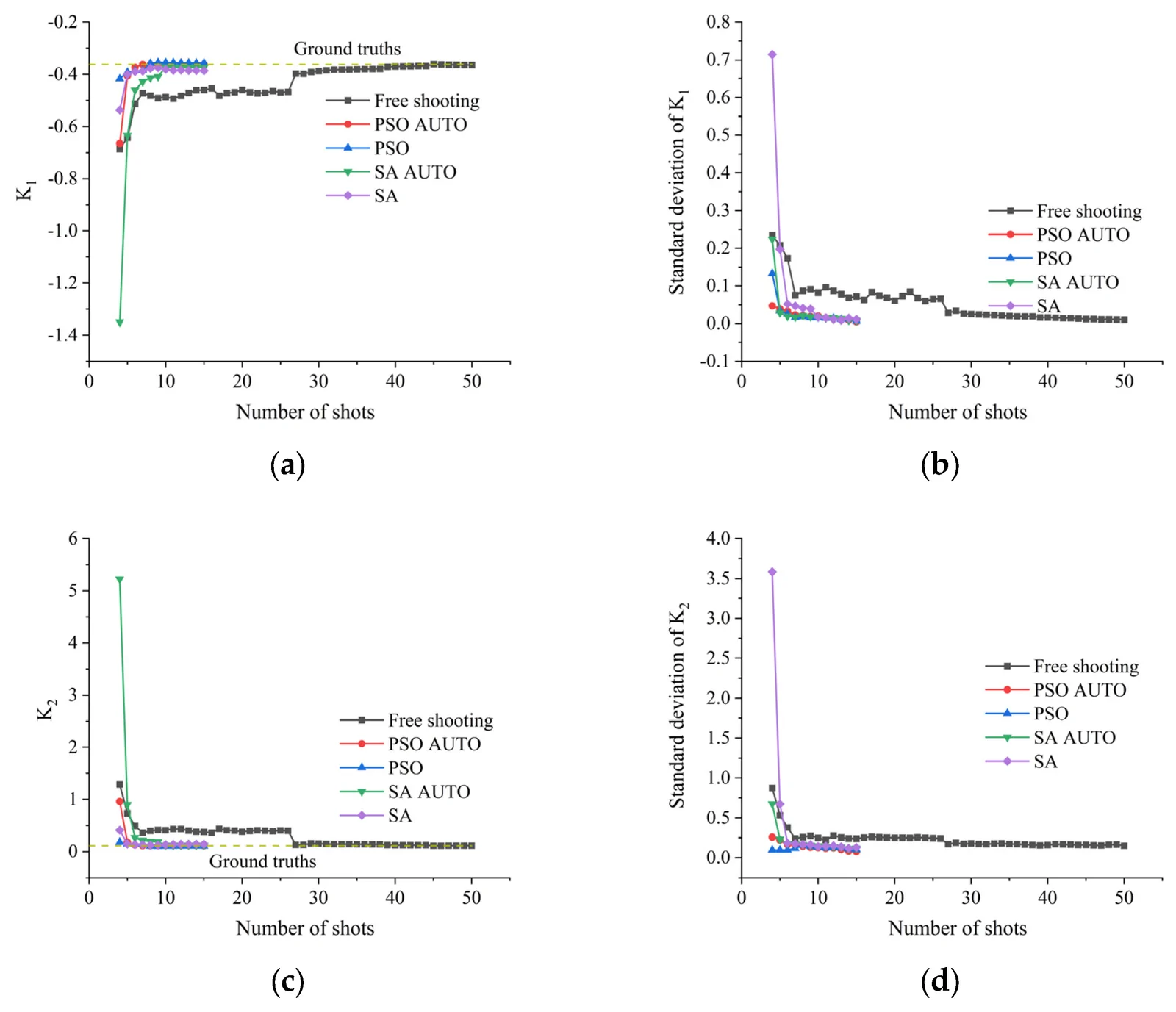
Calibration results of distortion parameters $k_{1}$ and $k_{2}$ of the wide-angle camera under different schemes. (**a**) Calibration results of $k_{1}$ of the wide-angle camera under different schemes; (**b**) standard deviation of calibrated $k_{1}$ of the wide-angle camera under different schemes; (**c**) calibration results of $k_{2}$ of the wide-angle camera under different schemes; (**d**) standard deviation of calibrated $k_{2}$ of the wide-angle camera under different schemes.

The lens of wide-angle cameras had large value of distortion, so the effect of distortion correction exerted important influences on the overall effect of parameter calibration of the cameras. In this study, the distortion values $k_{1}$ and $k_{2}$ of the wide-angle camera under different schemes were analyzed, as shown in Figure 8. It can be found in Figure 8a,c that PSO AUTO obtained the calibration results closest to the real distortion value of the wide-angle camera, with fewer calibration images. In Figure 8b,d, from the changes of the standard deviation of distortion coefficient of the wide-angle camera, it tended to decrease in all three methods, including free-shooting, SA AUTO, and PSO AUTO. However, the standard deviation of the calibrated distortion coefficient of PSO AUTO decreased the fastest and was closest to zero. It demonstrates that PSO AUTO could improve the calibration accuracy of distortion parameters of wide-angle cameras.

#### 6.2.3. The Calibration Results of the Ultra-Wide-Angle Camera (FOV ≈ 180°)

To clarify the applicable boundary conditions of the proposed algorithm, supplementary experiments are conducted on a fisheye camera with a field of view (FOV) of 180° (Model: ELP-USB8MP02G-BL180, equipped with Sony IMX179 sensor and 8MP resolution). The horizontal, vertical and diagonal field of view of the camera all reach 180°, which can effectively verify the performance of the algorithm under the extreme conditions of the ultra-wide-angle.

As shown in Table 6, under the extreme ultra-wide-angle conditions, the PSO AUTO method still achieves better calibration performance than the free-shooting method and the SA AUTO method. The reprojection error obtained by the proposed PSO AUTO method is 2.683, which is reduced by 44.9% compared with 4.872 of the free-shooting method and reduced by 21.4% compared with 3.415 of the SA AUTO method. The focal length-estimation error is 4.28 mm, which is reduced by 65.6% compared with 12.46 mm of the free-shooting method and reduced by 47.5% compared with 8.15 mm of the SA AUTO method. The distortion center-offset error of the PSO AUTO method is 6.2, which is reduced by 75.8% compared with 25.6 of the free-shooting method and reduced by 58.1%, compared with 14.8 of the SA AUTO method.

Compared with the results under FOV ≈ 150°, the absolute accuracy of the proposed PSO AUTO method decreases under the condition of FOV = 180°, with the reprojection error increasing from 1.75 to 2.68 and the focal length error rising from 1.15 mm to 4.28 mm. This phenomenon is in line with expectations. The projection model of the fisheye lens extends from the radial distortion of the pinhole model to highly nonlinear projection forms such as the equidistant projection (*r_d_* = *fθ*), which significantly increases the degree of distortion nonlinearity. Nevertheless, the relative superiority of the proposed PSO AUTO method over the comparison methods is not weakened but further enlarged, which fully verifies the robustness and adaptability of the algorithm under extreme ultra-wide-angle conditions.

The regional error analysis (Table 7) indicates that for the proposed PSO AUTO method, the reprojection error in the edge region (3.28) is significantly higher than that in the central region (1.24), with a difference of 2.65 times. This discrepancy is more prominent for the free-shooting method, with a difference of 3.43 times. The above results demonstrate that the proposed distortion pre-estimation module is particularly effective for distortion correction in edge regions. In terms of convergence speed, the proposed PSO AUTO method achieves stable calibration accuracy with an average of 7.8 images, which is substantially better than the SA AUTO method (10.2 images) and the free-shooting method (14.5 images). The total prediction time of the PSO AUTO method is 168.7 s. Although it is slightly higher than 145 s obtained under FOV ≈ 150° due to the larger parameter space of the fisheye distortion model, it is still much more efficient than the SA AUTO method (986.4 s), achieving approximately 5.8 times speedup.

Combining three groups of experiments with FOV ≈ 60° (ordinary camera), FOV ≈ 150° (wide-angle camera), and FOV = 180° (ultra-wide-angle fisheye camera), the applicable boundary of the proposed algorithm can be clearly defined. For cameras with FOV ≤ 150°, the algorithm achieves optimal performance with a reprojection error lower than 1.75 pixels. For ultra-wide-angle cameras with 150° < FOV ≤ 180°, the algorithm remains valid with a reprojection error below 2.68 pixels, although the calibration accuracy decreases slightly. For extreme fisheye lenses with FOV > 180°, the performance of the algorithm requires further verification.

#### 6.2.4. The Calibration Experiment Under Complex Light Setting

To verify the robustness of the proposed algorithm under complex light settings, supplementary experiments are conducted under three typical light settings: normal light (indoor fluorescent lamp, 300–500 lux), strong light (an additional 500 W fill light directly irradiating the calibration board, 2000–3000 lux), and low light (ambient light only, less than 50 lux). The experiments are performed on an ordinary camera (Logitech C270i) and a wide-angle camera (VISHINSGAE X30), with 30 conducted trials implemented for each condition.

Table 8 and Table 9 present the calibration results of the ordinary camera and wide-angle camera under different light settings, respectively. The experimental results show that under strong-light settings, highlight reflection on the surface of the calibration board reduces corner-detection accuracy, leading to varying degrees of degradation in the calibration accuracy of all methods. Under low-light settings, the signal-to-noise ratio of images decreases, which increases the difficulty of corner detection and further deteriorates the calibration accuracy. Nevertheless, the proposed PSO AUTO method outperforms the free-shooting method and the SA AUTO method under all light settings, and its superiority is more prominent under extreme light conditions.

Specifically, for the ordinary camera, the reprojection errors achieved by the proposed PSO AUTO method under normal-, strong-, and low-light settings are 0.1599, 0.1642, and 0.1718, respectively. Compared with the free-shooting method, the accuracy improvements of the proposed PSO AUTO method under the three light settings reach 6.6%, 23.8%, and 35.1%. For the wide-angle camera, the corresponding improvement rates are 23.8%, 33.0%, and 38.3%. The final reprojection error of 1.7465 obtained with the pre-estimation module is identical to the reprojection error of PSO AUTO (3 free + 12 guidance) listed in Table 4, which verifies the consistency of experimental data.

It is worth noting that under low-light settings, the corner-detection success rate of the free-shooting method drops to 78.6%, which seriously undermines the calibration reliability. In contrast, the proposed PSO AUTO method maintains a corner-detection success rate of 88.2%, by virtue of the optimized poses recommended by the guidance system. The pose guidance strategy effectively avoids corner blurring caused by extreme shooting angles, and keeps the calibration board corners within the optimal imaging region of the camera. Therefore, stable and clear corner responses can be obtained even under low-light settings.

In terms of calibration-time consumption (Table 10), the total prediction time of the proposed PSO AUTO method increases to 152.6 s and 168.4 s under strong -and low-lighting conditions, respectively (145.0 s under normal lighting). This is mainly due to the decreased confidence of corner detection, which requires the guidance system to perform more rounds of optimization to ensure calibration accuracy. The reduced corner-detection success rate under low-lighting conditions increases the number of retries and optimization iterations in each calibration process, thereby prolonging the total prediction time. Nevertheless, the accuracy improvement brought by this slight time cost is worthwhile, with a 38.3% accuracy gain achieved under low-lighting conditions.

The above results have fully demonstrated that the proposed PSO AUTO calibration guidance system exhibits good stability and robustness under diverse lighting conditions.

#### 6.2.5. The Comparison Between Recent Calibrations

To further demonstrate the superiority of the proposed method over state-of-the-art techniques, Table 11 conducts a qualitative comparison between our algorithm and recent approaches including IGAPSO [57], the improved gray wolf genetic algorithm [58], Deep-BrownConrady [59], and GeoCalib [60,61] from multiple dimensions: methodology, supported camera types, required number of images, training data requirement and reprojection error.

It should be noted that all the competing methods adopt distinct experimental platforms, camera models, and calibration scenarios. Hence, the reprojection error values listed in the table are for reference only, and cannot be used for rigorous horizontal quantitative comparison. Even so, Table 11 can clearly reveal the discrepancies in methodological frameworks and applicable scenarios among different calibration methods.

As is showed in Table 11, the methods based on Swarm Intelligence, IGAPSO and the improved grey wolf genetic algorithm are most similar to the method applied by this paper. However, both methods are only applicable to ordinary cameras and require a larger number of calibration images to obtain stable optimization results. In contrast, our method, through the variable-weight PSO strategy and the distortion pre-estimation module, supports wide-angle cameras while achieving faster convergence and higher calibration accuracy.

Deep learning-based methods (Deep-BrownConrady and GeoCalib) have the potential to estimate camera parameters from a single image, but their performance heavily depends on the quality and scale of training data, and their generalization capability across different camera models is limited. In contrast, our method requires no training data and can achieve high calibration accuracy through online optimization, thereby offering greater flexibility and interpretability in practical deployment.

### 6.3. Real-World Evaluation

Although the effectiveness of the guidance algorithm in this study has been evaluated contrastively in the comprehensive evaluation, it is hoped that the effectiveness of its proposed next optimal pose can be evaluated in a real-world example. To this end, two kinds of trials were designed and the results were compared with images freely shot and shot with SA AUTO. It is hard to evaluate the results of camera calibration because of the difficulty of obtaining the ground-truths. Therefore, the study designed two kinds of trials to evaluate the effect of camera calibration by evaluating the results in application, including pose estimation and reconstruction based on point clouds.

#### 6.3.1. Real-World Evaluation of the Calibration of Ordinary Cameras

Firstly, a set of quantitative trials were designed to compare the Euclidean distance errors of each corner point in the calibration image, so as to compare the effects of different calibration algorithms in ordinary camera calibration.

By means of MATLAB calibration toolbox [62], the calibration results obtained by different calibration algorithms could be saved. Then checkerboard images collected by the same group were calibrated with the usage of the intrinsics calibrated by different algorithms, so as to obtain the mean and standard deviation of Euclidean distance errors of corner points in each image.

The study firstly took three photos of the checkerboard calibration target, and then carried out calibration trials with 12 photos of the checkerboard calibration target taken by the designed system. Thirty rounds of trials were conducted and their results were averaged, as shown in Table 12. It can be found that both PSO AUTO and SA AUTO could achieve smaller mean of Euclidean distance error than that of 50 calibration images freely shot, under the condition of the same three initial calibration images. Moreover, the effect of PSO AUTO was better than that of SA AUTO.

In order to show that the guidance system was effective in reducing the Euclidean distance error, the study carried 30 rounds of trials, averaged their results, and compared mean and standard deviation of Euclidean distance error of each corner point obtained by the guided calibration and 20 images freely calibrated. The results are shown in Table 13. It can be found that the result of PSO AUTO after completing two images of calibration was better than that of 20 images freely calibrated by the user. The effect of SA AUTO was merely better than that of 10 images freely calibrated by the user. The reason might be that SA performed not as well as PSO in solving nonlinear optimization. As a result, the angle of images taken under the guidance of SA was too large, which made it difficult for the user to calibrate completely, according to the guidance results.

The study compared reprojection error and prediction time of the ordinary camera through 50 images taken by three methods, including free shooting, PSO, SA, PSO AUTO and SA AUTO. Thirty rounds of trials were carried out and their results were averaged, as shown in Table 14. It can be found that the initial reprojection error obtained by the guidance system for camera calibration was 0.203611 (the result obtained after 3-free shooting), which was reduced to 0.159867 after PSO AUTO was used. The value was much lower than the reprojection error obtained by the user’s free calibration and SA AUTO. At the same time, the total prediction time used by PSO AUTO was much less than that used by SA AUTO. The table fully demonstrates that compared with SA AUTO, PSO AUTO significantly improved the calibration effect and running time.

**Figure 9:**
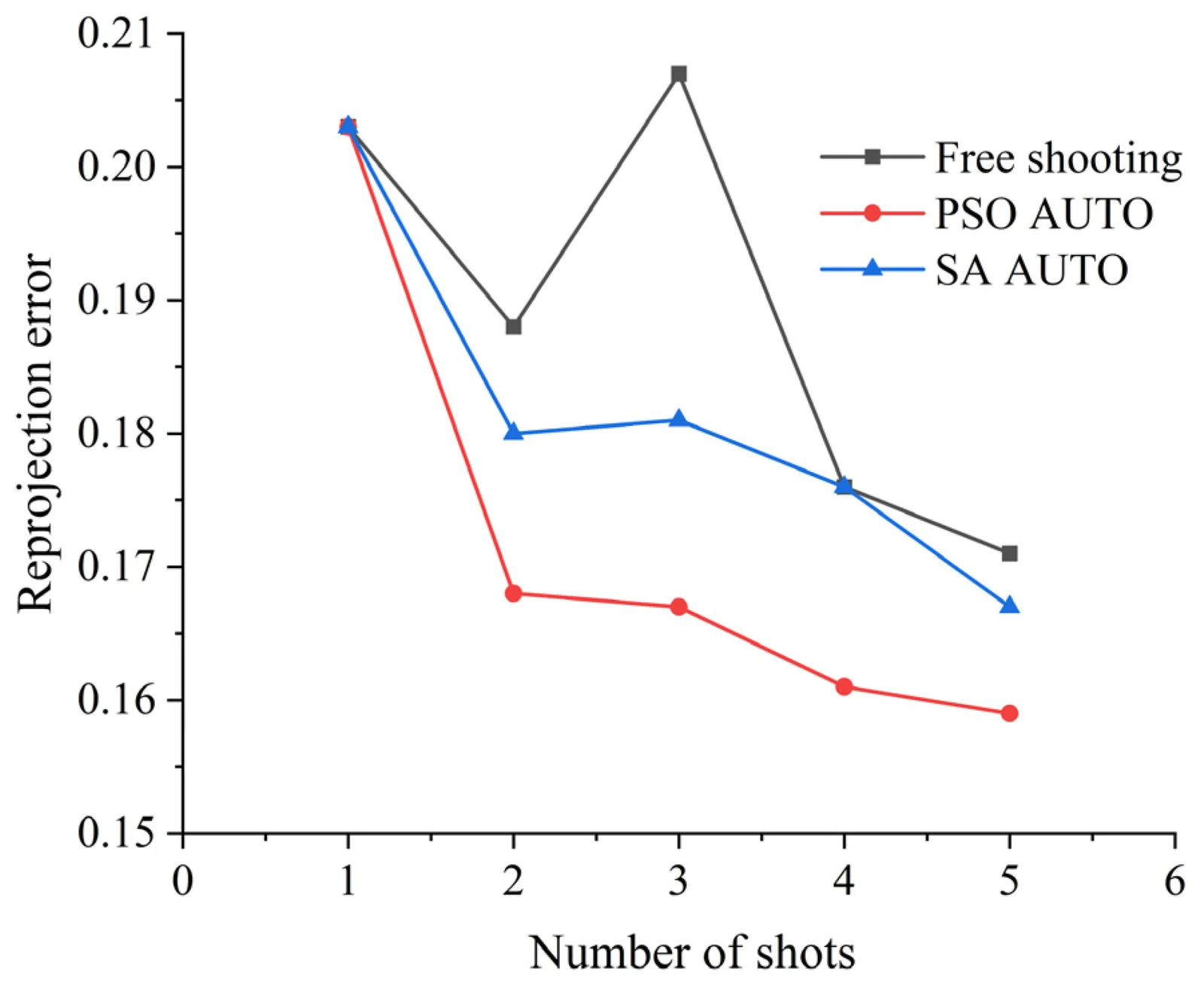
Reprojection error of the guidance system for camera calibration under different prediction methods.

The study compared reprojection error of the guidance system for calibration of the ordinary camera under three methods, including the user’s free shooting, PSO AUTO, and SA AUTO. Thirty rounds of trials were carried out and their results were averaged, as shown in Figure 9. It can be found that the reprojection error obtained by the PSO AUTO had the best optimization effect and decreased the fastest, compared with the results obtained by the SA AUTO and the user’s free shooting.

#### 6.3.2. Real-World Evaluation of Calibration of Wide-Angle Cameras

Consistent with the ordinary camera, the study carried out 30 rounds of trials and averaged their results. Through quantitative comparison of Euclidean distance error, the results of different calibration schemes were analyzed, as shown in Table 15.

It can be seen from the table that the PSO AUTO had the smallest Euclidean distance error and it tended to decrease continuously, proving to be the best effect. It was worth noting that the Euclidean distance error of SA AUTO showed an upward trend after the ninth image was taken. The reason was that SA AUTO sometimes predicted a large angle for the placement of calibration images, which made it difficult for the user to allow the actual and recommended placement to coincide, resulting in an increase of Euclidean distance error.

Thirty rounds of trials were carried out for wide-angle camera calibration and the results were average, as shown in Table 16. It can be seen that the introduction of distortion correction could optimize the calibration results when free shooting calibrated wide-angle cameras. The Euclidean distance error of PSO AUTO and SA AUTO after two guided shots were smaller than that of the user’s 20 free shots. Also, the effect of PSO AUTO was better than that of SA AUTO. However, in Table 15, both PSO AUTO and SA AUTO show an increase in the error after two guided shots, indicating that this phenomenon was inevitable. The guidance system aims to predict the possible result of the minimum Euclidean distance error, according to the images taken by the user. Even if there is a temporary small increase in the error, it can remove the influence in the subsequent calibration. In contrast, the result of the user’s free shooting brims with the unknown and unpredictability.

Table 17 shows the reprojection error of calibration of the wide-angle camera with its own guidance system. The result of PSO AUTO was still better than that of the user’s free calibration and SA AUTO. Its prediction time was much lower than that of SA AUTO.

The study compared reprojection error and prediction time of the wide-angle camera through 50 pictures taken by three methods, including the user’s free shooting, PSO AUTO and SA AUTO. Thirty rounds of trials were carried out and their results were averaged, as shown in Table 17. It can be found that the PSO AUTO achieved the optimal effect of the reduction of reprojection error, and its consumed time was much lower than that of other methods.

**Figure 10:**
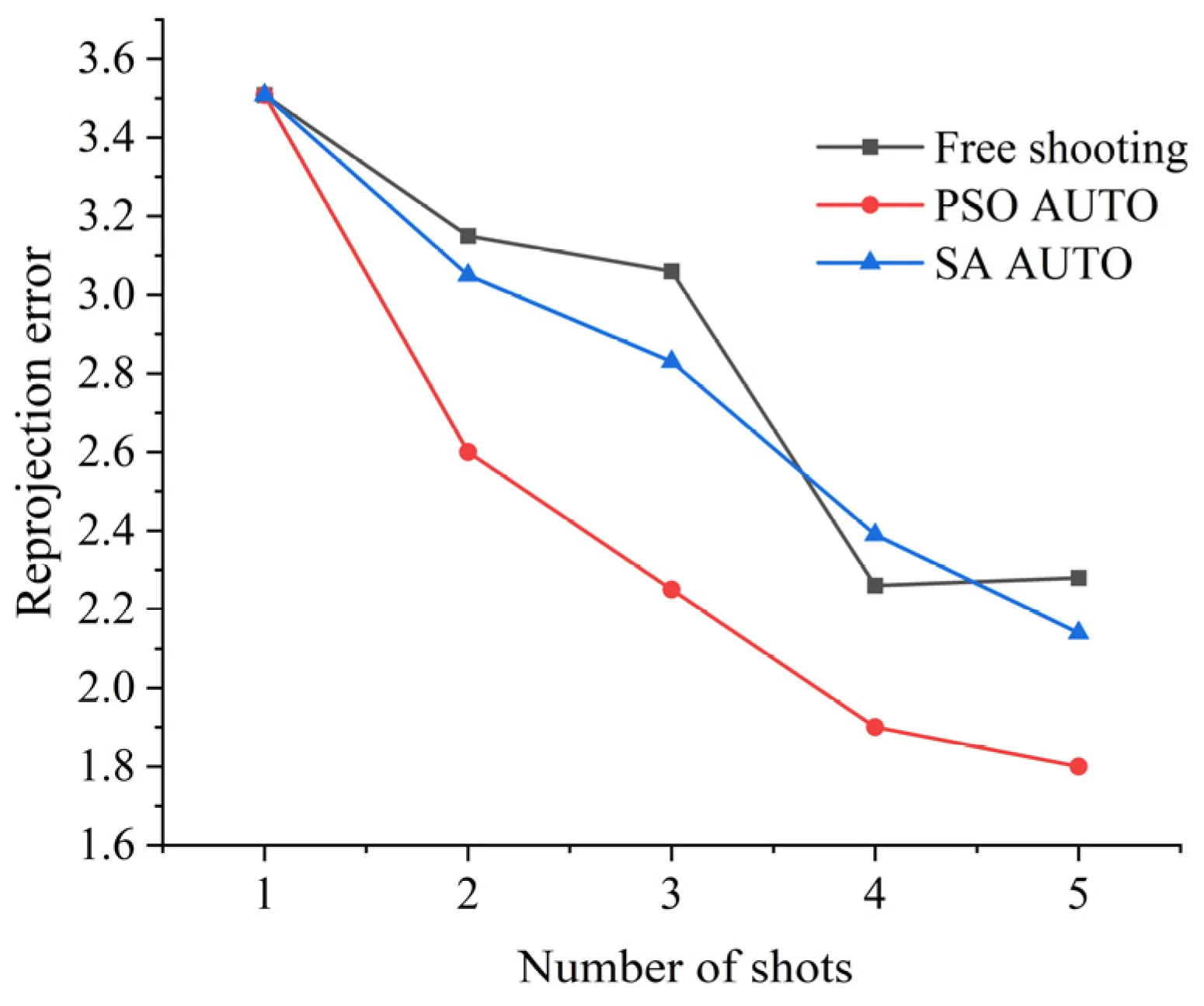
Comparison of reprojection error under different calibration methods.

The study compared the reprojection error of guidance system for the wide-angle camera under three methods, including the user’s free shooting, PSO AUTO and SA AUTO. Thirty rounds of trials were carried and their results were averaged, as shown in Figure 10. It can be found that the reprojection error obtained by PSO AUTO had the best optimization effect and its reprojection error decreased the fastest, compared with the results obtained by SA AUTO and the user’s free shooting.

#### 6.3.3. 3D Reconstruction Based on Point Clouds of Ordinary Cameras

**Figure 11:**
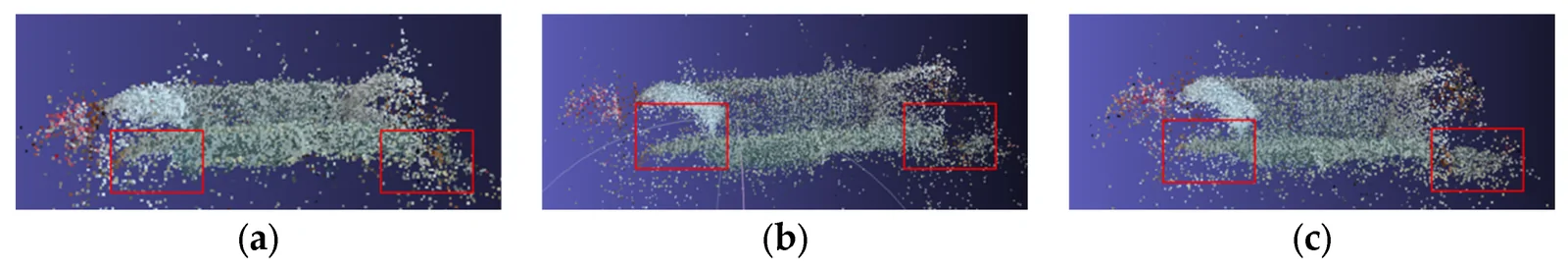
Effect of reconstruction based on point clouds. (**a**) Reconstruction of parameters obtained by 20 images of free shooting; (**b**) reconstruction of parameters obtained by three images of free shooting and four images taken with PSO AUTO; (**c**) reconstruction of parameters obtained by three images of free shooting and four images taken with SA AUTO.

In this study, the ordinary camera was used to shoot 36 images around the object. Camera parameters obtained by three methods, including free shooting of 20 images, PSO AUTO and SA AUTO were involved to conduct reconstruction based on point clouds in visual SFM, as shown in Figure 11.

It can be seen from the above reconstruction that all the three methods could reconstruct the basic shape of the original object. The parameters obtained by PSO AUTO were better than that of SA AUTO in the reconstruction of point clouds, and the method was able to reconstruct more detailed features in the area circled by the red boxes. The reconstruction based on parameters obtained by free shooting yielded the worst results, even with ill-defined edges of objects and distorted object shapes.

## 7. Discussion

This study proposed particle swarm optimization with autocorrelation matrices considered (PSO AUTO) for ordinary and wide-angle cameras. The new method could guide any user to complete calibration of various camera models. Meanwhile, the efficiency and accuracy of calibration for wide-angle cameras were further improved through distortion correction of a single image based on convex optimization. Trials suggested that the guidance system could obtain better calibration with fewer calibration images and shorter calculation time. In addition, the study designed two approaches, including fixed cameras with moving calibration targets, and moving cameras with fixed calibration targets, which met the demand of calibration for various cameras in film and television shooting. The completion of the system was able to significantly reduce errors of camera parameters in the process of film and television shooting, lower the cost of camera calibration, and save the time needed for equipment debugging, thus promoting the development of the film and television industry.

In terms of the applicable boundary of the proposed algorithm, systematic comparisons are conducted across three experimental groups with FOV ≈ 60° (ordinary camera), FOV ≈ 150° (wide-angle camera), and FOV = 180° (ultra-wide-angle fisheye camera). The experimental results reveal that the absolute accuracy of the proposed PSO AUTO method decreases when the FOV expands from 150° to 180°, with the reprojection error increasing from 1.75 to 2.68. This degradation is mainly attributed to the fact that the imaging model of fisheye lenses shifts from the radial distortion-based pinhole model to highly nonlinear projection modes, including the equidistant projection ($r_{d}=f\theta$) and equisolid-angle projection ($r_{d}=2fsin\left(\theta /2\right)$), which lead to a more complex distortion parameter space. Nevertheless, the relative superiority of the PSO AUTO method over the free-shooting method and the SA AUTO method becomes more prominent at FOV = 180°, demonstrating the excellent adaptability of the proposed algorithm to ultra-wide-angle distortion. Accordingly, this algorithm is recommended for scenarios with FOV ≤ 150°. For ultra-wide-angle cameras with 150° < FOV ≤ 180°, the algorithm is applicable under non-extreme accuracy requirements. For extreme fisheye lenses with FOV > 180°, further research integrating advanced and sophisticated fisheye imaging models is required to improve the calibration performance.

The proposed PSO AUTO method also exhibits favorable robustness under complex light settings (Section 6.2.4). As summarized in Table 8 and Table 9, under strong-light settings (2000–3000 lux), the reprojection errors of the proposed PSO AUTO method increase slightly from 0.1599 (ordinary camera) and 1.7465 (wide-angle camera) under normal light to 0.1642 and 1.8932, respectively. Under low-light settings (<50 lux), the reprojection errors rise moderately to 0.1718 and 2.1086. In contrast, the free-shooting method suffers a dramatic accuracy degradation under low-light conditions. Its reprojection errors increase substantially from 0.1712 (ordinary camera) and 2.2904 (wide-angle camera) under normal light to 0.2648 and 3.4182, with a growth rate of 54.7% and 49.2%, respectively. These results indicate that the optimized poses recommended by the guidance system effectively mitigate the adverse effects of extreme illumination on calibration accuracy, enabling the proposed PSO AUTO method to possess significantly better environmental adaptability than the free-shooting method.

In terms of computational complexity, as presented in the Section Ablation Study: Comparison Between Standard PSO and Variable-Weight PSO and Table 3, the single prediction time of the proposed PSO AUTO method is 7.25 s, which is significantly lower than approximately 60 s required by the SA AUTO method. For the complete calibration procedure utilizing three initial images and 12 guided images, the total prediction time of the proposed PSO AUTO method is 145.0 s, accounting for roughly one-sixth of the 918.2 s consumed by the SA AUTO method (Table 6). Even under the ultra-wide-angle condition of FOV = 180°, the total prediction time of the PSO AUTO method remains only 168.7 s, which is still far more efficient than 986.4 s of the SA AUTO method (Table 6).

Compared with recent swarm intelligence optimization-based calibration methods, such as IGAPSO [57] and the improved grey wolf genetic algorithm [58], the proposed method exhibits distinct advantages in multiple aspects. First, the variable-weight PSO strategy adopts a linearly decreasing inertia-weight mechanism. It utilizes a large inertia weight for global search at the early iteration stage and a small inertia weight for local refinement at the late stage, which effectively avoids the local optimum problem inherent in standard PSO. In contrast, although IGAPSO integrates genetic algorithm and PSO frameworks, it adopts fixed weight parameters and lacks an adaptive global-local search balancing mechanism. Second, the proposed method embeds a dedicated distortion pre-estimation module to address the calibration challenges of wide-angle cameras, which has rarely been investigated in existing swarm intelligence-based calibration methods. Compared with deep-learning-based methods such as Deep-BrownConrady [59] and GeoCalib [61], the proposed method exhibits two merits: it requires no large-scale training data or costly annotation, and no retraining is needed for generalization across different camera models. Nevertheless, deep-learning-based methods possess remarkable advantages in inference speed. They can achieve millisecond-level estimation for a single image, making them well-suited for scenarios with stringent real-time requirements. By contrast, our method takes approximately 7.25 s per pose prediction, leaving room for improvement in real-time performance. Future work will explore combining deep-learning-based feature extraction with our optimization framework to further boost efficiency while preserving estimation accuracy.

In the future, it is planned to extend the method to other fields, such as binocular camera calibration in binocular vision reconstruction.

## 8. Conclusions

This paper proposes a camera calibration guidance system based on variable-weight particle swarm optimization (PSO). By automatically recommending the optimal next pose of the calibration board, the system effectively reduces uncertainties in the calibration process and achieves high-precision and high-efficiency camera parameter calibration. To address the low calibration accuracy caused by severe distortion in wide-angle camera calibration, this paper introduces a single-image distortion pre-estimation module based on convex optimization, which provides a new solution for acquiring effective distortion prior knowledge.

The core contributions of this paper can be summarized into three aspects. First, at the algorithm level, a linear decreasing inertia weight PSO strategy is proposed, where the inertia weight w decreases linearly from 0.9 to 0.4. Compared with the standard PSO algorithm (w = 1) and the simulated annealing algorithm, the proposed strategy substantially improves the global search capability and convergence speed. Ablation experiments demonstrate that the variable-weight PSO reduces the focal length-estimation error by 87.2%, decreases the reprojection error by 10.4%, and lowers the number of convergence iterations by 40.1%, compared with the standard PSO. Second, at the module level, a single-image distortion pre-estimation method based on convex optimization is designed to provide accurate initial distortion priors for wide-angle camera calibration. Experimental results show that after enabling the pre-estimation module, the estimation errors of distortion coefficients k_1_ and k_2_ are reduced by 88.9% and 88.2%, respectively, and the reprojection error decreases from 2.4231 to 1.7465. Third, at the system level, a fully automated calibration guidance workflow is implemented for both ordinary and wide-angle cameras. The proposed system can generate reliable pose recommendations without manual intervention, reducing the single prediction time from approximately 60 s of the SA method to 7.25 s of the proposed method.

Extensive experiments on ordinary cameras (FOV ≈ 60°) and wide-angle cameras (FOV ≈ 150°) verify that the proposed PSO AUTO method outperforms the free-shooting method and the SA AUTO method in terms of focal length estimation, distortion coefficient estimation, and reprojection error. With 12 guided images for calibration, the total prediction time of the proposed PSO AUTO method is 145 s, which is substantially better than 918 s of the SA AUTO method, achieving approximately 6.3 times speedup. Furthermore, three-dimensional point-cloud reconstruction experiments are conducted, to further validate the practical applicability of the calibrated parameters. The reconstruction results obtained by the PSO AUTO method exhibit richer detail preservation and higher shape accuracy. Supplementary experiments on the ultra-wide-angle fisheye camera (FOV = 180°) show that the proposed PSO AUTO method achieves a reprojection error of 2.683 pixels, which is reduced by 44.9% compared with 4.872 pixels of the free-shooting method and decreased by 21.4% compared with 3.415 pixels of the SA AUTO method. Although the absolute calibration accuracy declines under ultra-wide-angle conditions, the relative superiority of the proposed method is further enhanced. In addition, complex light setting experiments demonstrate that the PSO AUTO method maintains favorable calibration accuracy under both strong-light (2000–3000 lux) and low-light (<50 lux) conditions. Specifically, the accuracy improvement over the free-shooting method increases to 35.1% under extreme-low-light settings.

The proposed method also has several limitations. First, the algorithm performance is partially dependent on the physical qualities of the calibration board, including flatness, corner accuracy, and surface reflection characteristics. Second, the current distortion pre-estimation module mainly focuses on radial distortion and requires further extension for lenses with prominent tangential distortion. Third, the method is currently applicable only to monocular cameras, and has not been extended to binocular or multi-camera systems. Fourth, the corner-detection success rate still needs to be improved under extreme-low-light settings (<50 lux).

Future research will focus on the following directions: (1) extend the proposed framework to binocular and multi-camera calibration systems to realize the joint optimization of intrinsic and extrinsic parameters; (2) integrate adaptive image enhancement and robust corner-detection algorithms to improve calibration reliability under extreme light settings; (3) explore a hybrid optimization strategy that combines PSO with gradient-based local refinement, to further accelerate the convergence speed; (4) develop a lightweight version of the algorithm for deployment on embedded platforms; and (5) integrate deep learning-based feature-extraction methods and explore natural scene calibration without dedicated calibration boards. The proposed method can be widely applied to various visual tasks such as 3D reconstruction, visual perception, and film shooting, which can significantly reduce calibration errors and equipment debugging time.

## Figures and Tables

**Table 1:** Methodological comparison between the proposed method and the algorithm proposed by Peng et al. [12].

Comparison Dimension	Peng et al. [12] (SA)	Proposed Method (PSO AUTO)
Optimized Algorithm	Simulated annealing (SA)	Variable weight particle swarm optimization (PSO)
Global Searching Competence	Weak, prone to local optima, manual intervention required	Strong, linear decreasing weight strategy balances global and local search
Convergence Speed	Slow (approximately 60 s per time)	Fast (approximately 7.25 s per estimation)
Applicable Camera Type	Ordinary camera only	Ordinary camera+wide-angle camera
Distortion Processing	No special treatment	Single-image convex optimization for distortion pre-estimation
User Interaction	Manual intervention required to escape local optima	Fully automatic operation without manual intervention
Calibration Accuracy Improvement	Limited	Significant (reprojection error reduced by 10.4–27.9%)

**Table 2:** The key realizing parameters of PSO algorithm.

Parameter Name	Symbol	Value	Explanation
Number of Particles (Population Size)	N	30	Each particle represents a candidate pose hypothesis
Maximum Number of Iterations	$t_{max}$	200	One of the main termination conditions
Maximum Inertia Weight	$w_{max}$	0.9	Global search weight at initial iteration
Minimum Inertia Weight	$w_{min}$	0.4	Local search weight at final iteration
Cognitive learning factor	$c_{1}$	1.5	Cognitive acceleration coefficient
Social Learning Factor	$c_{2}$	1.5	Social acceleration coefficient
Position Boundary	$\left[X_{min},X_{max}\right]$	Dynamically set according to the calibration board workspace	Search space boundary for particle positions
Velocity Boundary	$\left[V_{min},V_{max}\right]$	±10% of position boundary	Velocity clamping range for particles
Convergence Criterion (Fitness Variation Threshold)	ε	1 × 10^−6^	Early termination if fitness variation is below this threshold
Initialization Strategy	—	Uniform random initialization within the feasible pose space	Ensures the diversity of initial group
Primary Termination Condition	—	$t\geq t_{max}$	Termination upon reaching the maximum number of iterations
Secondary Termination Condition	—	$\left|\Sigma_{t}-\Sigma_{t-1}\right|<\varepsilon$	Early termination when the change of fitness value is less than the threshold

**Table 3:** Comparative experimental results of standard PSO and variable-weight PSO.

Evaluation Index	Standard PS (w = 1)	Variable-Weight PSO (w: 0.9→0.4)	Improvement Rate
Focal Length Estimation Error (mm)	2.42	0.31	87.20%
$k_{1}$ Estimation Error	0.0044	0.0008	81.80%
$k_{2}$ Estimation Error	0.0037	0.0008	78.40%
Reprojection Error (pixel)	0.1784	0.1599	10.40%
Convergence Iterations	187	112	40.10%
Single Prediction Time (s)	8.64	7.25	16.10%
Probability of Falling into Local Optimum	5/30 (16.7%)	1/30 (3.3%)	80.00%

**Table 4:** Comparative experimental results of PSO AUTO with and without distortion pre-estimation.

Evaluation Indicator	Without Distortion Pre-Estimation	with Distortion Pre-Estimation	Improvement Rate
Focal Length Estimation Error (mm)	5.84	1.15	80.3%
k_1_ Estimation Error	0.027	0.003	88.9%
k_2_ Estimation Error	0.017	0.002	88.2%
Reprojection Error (pixel)	2.4231	1.7465	27.9%
Distortion Center-Offset Error(pixel)	11.6	0.8	93.1%
The images needed	9.8	6.2	36.7%
Total estimation time (s)	139.8	145	−3.7%

**Table 5:** Regional distortion pre-estimation analysis.

Image Region	Definition	Without Pre-Estimation	with Pre-Estimation	The Rate of Error Reduction
Central Region	Radius ≤ Short side of the image× 1/3	0.38 pixel	0.28 pixel	26.3%
Edge Region	Radius > Short side × 1/3	2.86 pixel	1.15 pixel	59.8%
The Average of the Whole Image	—	1.96 pixel	0.82 pixel	58.2%

**Table 6:** Comparison results of ultra-wide-angle camera (fov = 180°) with different calibration methods.

Evaluation Indicators	Free Shooting (20 Pieces)	SA AUTO (3 + 12 Pieces)	PSO AUTO (3 + 12 Pieces)	PSO AUTO vs. Free Shooting	PSO AUTO vs. SA AUTO
Focal Length Estimation Error (mm)	12.46	8.15	4.28	65.6%	47.5%
k_1_ Estimation Error	0.084	0.052	0.025	70.2%	51.9%
k_2_ Estimation Error	0.112	0.068	0.032	71.4%	52.9%
Reprojection Error (pixel)	4.872	3.415	2.683	44.9%	21.4%
Distortion Center-Offset Error (pixel)	25.6	14.8	6.2	75.8%	58.1%
The images needed	14.5	10.2	7.8	46.2%	23.5%
Total estimation time (s)	—	986.4	168.7	—	82.9%

**Table 7:** Regional reprojection error analysis of ultra-wide-angle camera (FOV = 180°).

Image Region	Definition	Free Shooting	SA AUTO	PSO AUTO	The Error Decrease Rate of PSO AUTO (vs. Free Shooting)
Central Region	Radius ≤ Short side of the image × 1/3	1.86	1.52	**1.24**	33.3%
Edge Region	Radius > Short side of the image × 1/3	6.38	4.36	**3.28**	48.6%
The Average of the Whole Image	—	4.87	3.42	**2.68**	44.9%

**Table 8:** Comparison of reprojection errors of ordinary camera under different lighting conditions.

Light Settings	Illumination Intensity	Free Shooting (20 Pieces)	SA AUTO (3 + 12 Pieces)	PSO AUTO (3 + 12 Pieces)	Improvement in PSO AUTO (vs. Free Shooting)
Normal Light	300–500 lux	0.1712	0.1673	0.1599	6.6%
Strong Light	2000–3000 lux	0.2156	0.1864	0.1642	23.8%
Low Light	<50 lux	0.2648	0.1975	0.1718	35.1%

**Table 9:** Comparison of reprojection errors of wide-angle camera under different lighting conditions.

Light Settings	Illumination Intensity	Free Shooting (20 Pieces)	SA AUTO (3 + 12 Pieces)	PSO AUTO (3 + 12 Pieces)	Improvement in PSO AUTO (vs. Free Shooting)
Normal Light	300–500 lux	2.2904	2.1839	1.7465	23.8%
Strong Light	2000–3000 lux	2.8247	2.4156	1.8932	33.0%
Low Light	<50 lux	3.4182	2.5628	2.1086	38.3%

**Table 10:** Comparison of corner-detection success rate and calibration-time consumption under different light settings.

Light Settings	Corner-Detection Success Rate (Free Shooting)	Corner-Detection Success Rate (Guided Shooting)	Average Time Consumption Per Single Calibration (Free Shooting)	Average Time Consumption Per Single Calibration (PSO AUTO)
Normal Light	99.20%	99.50%	6.8 s	145.0 s
Strong Light	92.50%	96.80%	8.3 s	152.6 s
Low Light	78.60%	88.20%	12.5 s	168.4 s

**Table 11:** Comparison of the proposed method with recent calibration methods.

Dimension of Comparison	Improved Grey Wolf Genetic Algorithm [58]	Deep-Brown Conrady [59]	GeoCalib [61]	GeoCalib [61]	Proposed Method
Types of method	Improved grey wolf genetic algorithm	Deep learning (ResNet)	Deep learning + geometric optimization	Deep learning + geometric optimization	Variable weight
					PSO
Supported camera types	Ordinary camera	Ordinary camera	Ordinary camera	Ordinary camera	Normal+
					wide-angle
The number of images needed	Multiple images	Single image	Single image	Single image	3–15 pieces
Whether training data is required	No	Yes (Composite Dataset)	Yes	Yes	No
Distortion processing	Nonlinear optimization	Nonlinear optimization	Brown–Conrady model	Implicit	Convex optimization pre-estimation
Reprojection error (reference value)	—	0.02054 px	—	—	0.1599 px (Ordinary)/1.7465 px (Wide-angle)
Whether supported guided calibration	No	No	No	No	Yes

**Table 12:** Mean and standard deviation of Euclidean distance errors of corner points under different methods (with 4–12 Guided Images).

Free Calibration	Mean	Std	PSO AUTO	Mean	Std	SA AUTO	Mean	Std
3-free	1.54262	1.56262	3-free + 4 guidance	1.30382	1.35859	3-free + 4 guidance	1.43449	1.34747
10-free	1.46314	1.42723	3-free + 6 guidance	1.28356	1.36336	3-free + 6 guidance	1.37724	1.45151
20-free	1.30278	1.32765	3-free + 9 guidance	1.27760	1.34545	3-free + 9 guidance	1.33984	1.44461
50-free	1.27026	1.31549	3-free + 12 guidance	**1.19995**	**1.3025**	3-free + 12 guidance	1.23303	1.3384

**Table 13:** Mean and standard deviation of Euclidean distance errors of corner points under different methods (with 1–2 Guided Images).

Free Calibration	Mean	Std	PSO	Mean	Std	PSO AUTO	Mean	Std
3-free	3.04036	3.08345	3-free + 1wizard	2.8946	2.9663	3-free + 1wizard	2.67262	2.7750
10-free	2.83924	2.81786	3-free + 2wizard	2.7947	2.7485	3-free + 2wizard	2.5339	2.6793
20-free	2.55461	2.67671	—	—	—	—	—	—
			SA	mean	std	SA AUTO	mean	std
			3free + 1wizard	2.9168	3.01452	3free + 1wizard	2.78654	2.90315
			3free + 2wizard	2.8591	2.91031	3free + 2wizard	2.68888	2.79692

**Table 14:** Comparison of reprojection error and prediction time under different calibration methods.

OpenCV	Reprojection Error	PSO AUTO	Reprojection Error	SA AUTO	Reprojection Error
3-free	0.203611	3-free + 4-guidance	0.167864	3-free + 4-guidance	0.180089
10-free	0.188355	3-free + 6-guidance	0.167288	3-free + 6-guidance	0.180912
20-free	0.207203	3-free + 9-guidance	0.161078	3-free + 9-guidance	0.175514
50-free	0.171233	3-free + 12-guidance	**0.159867**	3-free + 12-guidance	0.167286
		**Total prediction time**	**121.420465 s**	**Total prediction time**	642.685841 s

**Table 15:** Prediction results of the wide-angle camera under different prediction methods.

	Mean	Std	PSO AUTO	Mean	Std	SA AUTO	Mean	Std
3-free	1.95781	3.25331	3-free + 4guidance	1.21037	1.02329	3-free + 4guidance	1.6389	2.60027
10-free	1.58739	1.51248	3-free + 6guidance	1.41014	1.37018	3-free + 6guidance	1.10402	2.06822
20-free	1.31931	1.0441	3-free + 9guidance	0.98122	0.95443	3-free + 9guidance	1.05707	0.89408
50-free	1.04699	0.93373	3-free + 12guidance	**0.96973**	**0.90342**	3-free + 12guidance	1.10971	0.93848

**Table 16:** Predicted error of the wide-angle camera under different prediction methods.

Free Shooting (Distortion Correction Excluded)	Mean	Std	Free Shooting (Distortion Correction Included)	Mean	Std
3-free	2.61271	3.11631	3-free	1.95781	3.25331
10-free	2.01567	1.18251	10-free	1.58739	1.51248
20-free	1.47355	1.47372	20-free	1.31931	1.0441
**PSO AUTO** **(Distortion Correction Included)**	**Mean**	**Std**	**SA AUTO** **(Distortion Correction Included)**	**Mean**	**Std**
3-free + 1-guidance	1.02496	0.99066	3-free + 1-guidance	1.12102	1.09575
3-free + 2-guidance	**0.93533**	**0.83913**	3-free + 2-guidance	1.10372	1.14179

**Table 17:** Prediction results and consumed time of the wide-angle camera under different methods.

OpenCV	Reprojection Error	PSO AUTO	Reprojection Error	SA AUTO	Reprojection Error
3-free	3.57672	3- free + 4-guidance	2.53411	3- free + 4-guidance	3.02577
10-free	3.13759	3-free + 6-guidance	2.23742	3-free + 6-guidance	2.80249
20-free	3.01632	3-free + 9-guidance	1.94072	3-free + 9-guidance	2.43923
50-free	2.29035	3-free + 12-guidance	1.7465	3-free + 12-guidance	2.18391
		Total prediction time	145.013347 s	Total prediction time	918.199938 s

## Data Availability

The original contributions presented in this study are included in the article. Further inquiries can be directed to the corresponding author.
